# Spatial transcriptomics uncovers the hybrid molecular identity, ciliated phenotype, and immune signature of adenomyosis lesions

**DOI:** 10.1126/sciadv.aea6379

**Published:** 2026-06-24

**Authors:** Alison Maclean, Emily Johnson, Corie Rushworth, Christopher J. Hill, Eva Caamaño Gutiérrez, Dharani K. Hapangama

**Affiliations:** ^1^ Department of Women’s and Children’s Health, Institute of Life Course and Medical Sciences, University of Liverpool, Liverpool, UK.; ^2^ Liverpool Women’s NHS Foundation Trust, Liverpool, UK.; ^3^ Computational Biology Facility, Liverpool Shared Research Facilities, University of Liverpool, Liverpool L69 7ZB, UK.; ^4^ Institute of Systems, Molecular and Integrative Biology, University of Liverpool, Liverpool L69 7ZB, UK.

## Abstract

Adenomyosis is a common gynecological condition characterized by ectopic endometrial-like tissue deep within the myometrium, causing debilitating symptoms. Treatment options are limited to hormones that primarily focus on symptom management only or invasive hysterectomy. Despite its prevalence, the pathogenesis of adenomyosis remains poorly understood, considerably impeding the development of targeted, true disease modifying treatments. Here, through spatial transcriptomics analysis of full-thickness human uterine wall biopsies (*n* = 10), we demonstrate that adenomyosis lesions exhibit a distinct transcriptomic profile, intermediate between matched endometrium and myometrium, characterized by enhanced epithelial ciliation and altered biological pathways. We reveal that adenomyosis lesion transcriptome more closely resembles the endometrial basalis than the functionalis, marked by enriched olfactory signaling, dysregulated oxidative phosphorylation and adenosine triphosphate synthesis, and an altered immune microenvironment indicative of chronic inflammation. In silico drug screening identified candidate compounds capable of reversing the adenomyosis lesion transcriptomic signature. These findings offer previously unknown insights into the molecular landscape of adenomyosis lesions and lay the groundwork for development of targeted, lesion-specific therapies that may preserve the eutopic endometrium and offer alternatives to hysterectomy.

## Teaser

Molecular profiling of adenomyosis lesions reveals targets for developing lesion-specific, uterus-sparing treatments.

## INTRODUCTION

Adenomyosis is a highly prevalent and debilitating gynecological disease affecting up to one in five women of reproductive age worldwide (*1*). Once thought to primarily affect women in their late reproductive years, recent advances in imaging modalities have revealed that it is also common among younger women who often wish to maintain their fertility (*2*). Characterized by the presence of ectopic endometrial-like tissue embedded within the myometrium, adenomyosis manifests as multicellular fibrotic lesions that give rise to heavy menstrual bleeding, severe dysmenorrhea, chronic pelvic pain, and subfertility (*3*, *4*). These symptoms exert a profound negative impact on physical health, emotional well-being, and quality of life, resulting in considerable social, economic, and health care burdens (*5*–*7*). Despite its high prevalence and far-reaching consequences, adenomyosis remains underdiagnosed and poorly understood, with its true pathogenesis yet to be fully elucidated (*8*, *9*). The clinical management of adenomyosis is hampered by this lack of mechanistic insight. Current treatment options are largely limited to hormonal therapies, which may provide symptomatic relief only and are often contraceptive (*10*), or to hysterectomy, which although curative, is invasive, life-altering, and unsuitable for women desiring future fertility. Gonadotropin-releasing hormone (GnRH) agonists induce a menopause-like state to treat adenomyosis but are often poorly tolerated due to hypoestrogenic side effects such as hot flushes, mood changes, and bone loss, limiting their suitability as a long-term treatment option. These can be mitigated with add-back estrogen and a progestin, although the latter may also cause intolerable side effects. Uterus-preserving surgical interventions, such as adenomyomectomy, are available in specialized centers but are not yet widely available and carry risks for subsequent pregnancies (*11*). The development of targeted disease-modifying therapies has been impeded by an incomplete understanding of how adenomyosis lesions arise, persist, and differ from the eutopic endometrium.

The human endometrium exhibits region-specific and cell type–specific functions throughout the menstrual cycle, with the superficial functionalis and deeper basalis layers showing distinct phenotypes and responses to ovarian steroid hormones (*12*, *13*). The basalis, regarded as the reservoir for progenitor or stem cells, is essential for regenerating the functionalis after each cycle (*14*–*17*). However, whether adenomyosis lesions, which comprise epithelial, stromal, immune, and endothelial cells within a fibrotic myometrial niche, display comparable regional diversity remains unclear. Fibrosis is a prominent feature of adenomyotic lesions, reflecting ongoing tissue injury and repair processes that contribute to lesion stiffness and disease progression (*18*, *19*). Multiple theories have been proposed to explain their formation, including invagination of the endometrial basalis and metaplasia of myometrial stem cells, but none have been definitively proven (*20*–*23*). Traditional molecular profiling approaches, such as bulk and single-cell RNA sequencing (RNA-seq), have provided important insights into endometrial biology (*24*, *25*) and the cellular composition of adenomyosis lesions (*16*, *26*). However, these techniques disrupt tissue architecture and lack the spatial resolution necessary to capture the intricate multicellular interactions and microenvironmental cues that underpin adenomyosis pathophysiology.

Spatial transcriptomics now enables high-resolution mapping of gene expression within intact tissue sections, preserving spatial context and cellular heterogeneity (*27*, *28*). This approach is uniquely suited to interrogate the molecular landscape of adenomyosis lesions in situ, facilitating comparative simultaneous analysis with matched eutopic endometrium, including both the functionalis and basalis subregions, within the same individual. Such analyses are essential for identifying lesion-specific molecular features and therapeutic targets. Here, we address the question of how adenomyosis lesions differ molecularly from the correctly located endometrium within the same uterus, using cell type–specific digital spatial profiling of full-thickness uterine wall biopsies. By preserving native tissue identity and cellular diversity, our study provides insights into the mechanisms driving adenomyosis lesion formation and maintenance and identifies candidate targets for the development of lesion-specific, uterus-sparing therapies. Our findings have the potential to transform the clinical management of adenomyosis, offering avenues for more effective and less invasive treatment options for millions of women worldwide.

## RESULTS

### Participant demographics

Ten women with symptomatic adenomyosis who underwent hysterectomy were included in this study. Histopathological examination of the resected uteri confirmed a diagnosis of intrinsic adenomyosis in all cases, defined as endometrial glands and stroma located in the inner third of the myometrium. All participants were in the secretory phase of the menstrual cycle and had not received hormonal treatments within the preceding three months. On the basis of histological dating within the secretory phase, samples were categorized as early (*n* = 1), mid (*n* = 4), and late (*n* = 5) secretory subphases. Uterine fibroids were present in 70% of participants and were classified as either intramural or subserosal; no submucosal fibroids were identified. Coexisting endometriosis was observed in 40% of cases. Sensitivity analysis indicated that variation in secretory subphase and coexisting fibroids or endometriosis did not measurably influence gene expression profiles or cellular composition across uterine compartments beyond the effects of adenomyosis (figs. S1 and S2). Participant demographics are presented in Table 1.

**Table 1.** Participant demographics. Endometriosis staging based on the revised ASRM classification (*104*). Fibroid subtypes classified by location on imaging or histology. BMI, body mass index; HMB, heavy menstrual bleeding; CPP, chronic pelvic pain.

	Median (range), or *n* (%)
Age (years)	44.3 (39–52)
BMI (kg/m^2^)	27.1 (21.5–31.7)
Smoking status	3/10 (30%)
Parity	1 (0–3)
Subfertility	2/10 (20%)
Adenomyosis subtype	Intrinsic: 10/10 (100%)
Gynecological comorbidities	
Endometriosis	4/10 (40%)
Stages I and II	3/4 (75%)*
Stages III and IV	1/4 (25%)*
Fibroids	7/10 (70%)
Intramural	7/7 (100%)*
Subserosal	1/7 (14%)*
Endometriosis and fibroids	4/10 (40%)
Symptoms	
HMB, dysmenorrhea, and CPP	3/10 (30%)
HMB and CPP	6/10 (60%)
CPP only	1/10 (10%)

* Percentages refer to proportion of patients with the subgroup.

### The adenomyosis lesion transcriptome resembles the endometrial basalis more than the functionalis

**Fig. 1.**
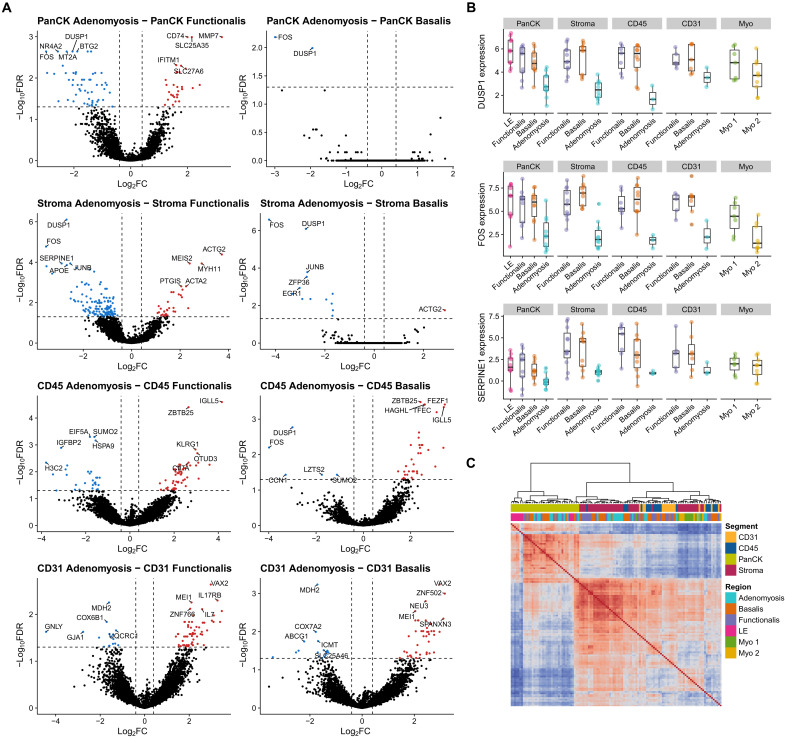
Differential gene expression between adenomyosis lesions and matched eutopic endometrial basalis, functionalis, and myometrium. (**A**) Volcano plots of DEGs between adenomyosis and eutopic endometrial functionalis and basalis subregions. *x* axis: log_2_ fold change (FC); *y* axis: −log_10_ FDR-adjusted *P* value. Red: up-regulated genes; blue: down-regulated genes; black: nonsignificant genes. Horizontal line: *P* < 0.05; vertical lines: log_2_ FC thresholds. Top row: epithelium (panCK+) DEGs (adenomyosis versus functionalis/basalis). Second row: stromal (panCK−, CD31−, CD45−) DEGs. Third row: leucocyte (CD45+) DEGs. Bottom row: endothelial (CD31+) DEGs. (**B**) Boxplots of key DEGs (*DUSP1*, *FOS*, *APOE*, *SERPINE1*, and *JUNB*) across adenomyosis lesion, eutopic endometrial functionalis, eutopic endometrial basalis, and myometrium; median shown by the center line, whiskers indicate the min/max. (**C**) Heatmap of gene expression across epithelial (panCK+), stromal, endothelial (CD31+), and immune (CD45+) cell populations isolated from eutopic endometrial functionalis, eutopic endometrial basalis, adenomyosis lesions, and adjacent myometrium. LE, luminal epithelium; Myo, myometrium.

Adenomyosis lesions displayed a distinct gene expression profile more closely resembling the matched endometrial basalis than the functionalis, consistent across all cell types but particularly pronounced in epithelial and stromal compartments (Fig. 1 and tables S1 to S10). The adenomyosis lesion signature was characterized by significant down-regulation of *FOS* and *DUSP1* in all cell types compared to endometrial basalis and functionalis (*P* < 0.01), suggesting altered regulation of cell fate processes. In adenomyosis lesion epithelium, stem cell marker *ALDH1A* was up-regulated (*P* < 0.0001), whereas *MT2A* and *BTG2* were down-regulated compared to functionalis (*P* = 0.002). Adenomyosis lesion stroma showed up-regulation of smooth muscle contraction genes (*ACTG2*, *MYH11*, and *ACTA2*; *P* < 0.001) and down-regulation of fibrinolysis inhibitor *SERPINE1* (*P* = 0.001). This revealed a lesion-specific transcriptomic profile that is distinct from the matched eutopic endometrium, with transcriptional divergence increasing with distance from the lesion site (Fig. 1 and tables S1 to S10).

### The transcriptomic profile of adenomyosis lesions lies intermediate between the matched endometrium and myometrium

**Fig. 2.**
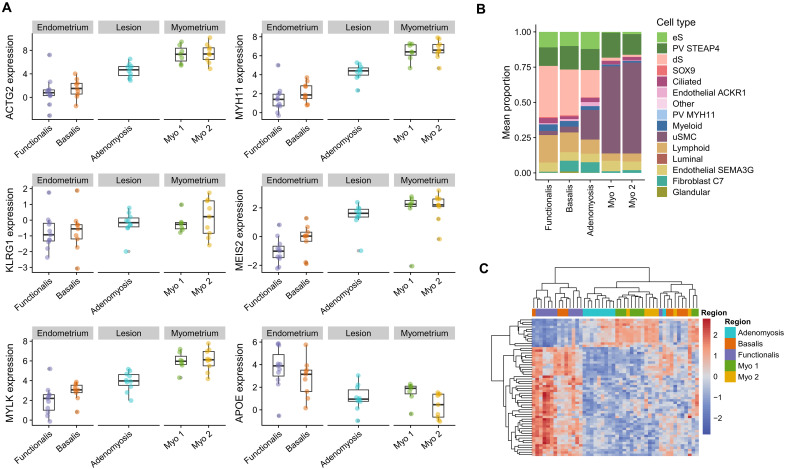
Transcriptional profile of adenomyosis lesions positioned between eutopic endometrium and myometrium. (**A**) Boxplots of key DEG expression (*ACTG2*, *MYH11*, *KLRG1*, *MEIS2*, *MYLK*, and *APOE*) in adenomyosis lesion stroma, eutopic endometrial functionalis stroma, eutopic endometrial basalis stroma, and myometrium; median and range shown. (**B**) Bar chart of stromal cell type proportions from deconvolution analysis in eutopic endometrial functionalis stroma, eutopic endometrial basalis stroma, adenomyosis lesion stroma, and myometrium. (**C**) Heatmap of pairwise Pearson correlation coefficients between stromal samples from eutopic endometrium (functionalis and basalis), adenomyosis lesions and myometrium. Correlations were calculated using the batch-corrected normalized expression data. Samples were hierarchically clustered on both rows and columns using the Ward D2 algorithm, with the dendrogram displayed for columns only, as clustering is symmetrical. The heatmap is annotated with segment and region metadata. The color scale represents the strength of sample-sample correlation. Myo, myometrium.

Adenomyosis lesions exhibited a well-defined transcriptomic profile, intermediate between the matched endometrium and myometrium, revealing a continuous gene expression gradient across these regions. This pattern is exemplified in the comparison between the adenomyosis lesion stroma, eutopic endometrial stroma, and myometrium, where key differentially expressed genes (DEGs) related to smooth muscle contraction and cell motility (e.g., *ACTG2*, *MYH11*, and *ACTA2*) display intermediate expression levels in lesions (Fig. 2A and tables S5 and S6). Gene sets relating to smooth muscle and muscle contraction were positively enriched in adenomyosis lesion stroma compared with endometrial basalis and functionalis stroma (table S11).

Cell deconvolution and differential abundance analysis revealed that, relative to functionalis stroma, adenomyosis lesion stroma contained more uterine smooth muscle cells (uSMCs) (*P* < 0.0001), more fibroblast C7 subtype (*P* = 0.0004), and fewer decidualized stromal cells (dSCs) (*P* = 0.0011), suggesting microenvironmental remodeling and uSMC inclusion in lesions. Compared to basalis stroma, adenomyosis lesion stroma exhibited increased uSMCs (*P* = 0.001). When compared to myometrium, adenomyosis lesion stroma had significantly fewer uSMCs (*P* < 0.0001) and a higher proportion of dSCs (*P* < 0.0001), endometrial stromal cells (eSCs) (*P* < 0.0001), myeloid cells (*P* < 0.0001), and fibroblast C7 subtype (*P* = 0.0004; Fig. 2 and tables S12 to S14).

Comparison of DEGs between the adenomyosis lesion stroma and adjacent myometrium demonstrated that these compartments remain transcriptionally distinct despite partial cellular overlap, and a direct comparison between Myo1 and Myo2 revealed extremely similar transcriptional profiles, with only one significant DEG, *CTR9* (fig. S3). Both regions were defined as within 400 μm of the endometrial-myometrial junction (Myo1) or the nearest adenomyosis lesion (Myo2), respectively. This intermediate gene expression pattern suggests that adenomyosis lesions have a hybrid identity, distinct from both their tissue of origin and their site of implantation. The identified intercellular communication pathways between myometrial cells and adenomyosis lesion components provide potential mechanisms for their growth and survival.

### Adenomyosis lesions display increased epithelial ciliation relative to endometrial basalis and functionalis subregions

**Fig. 3.**
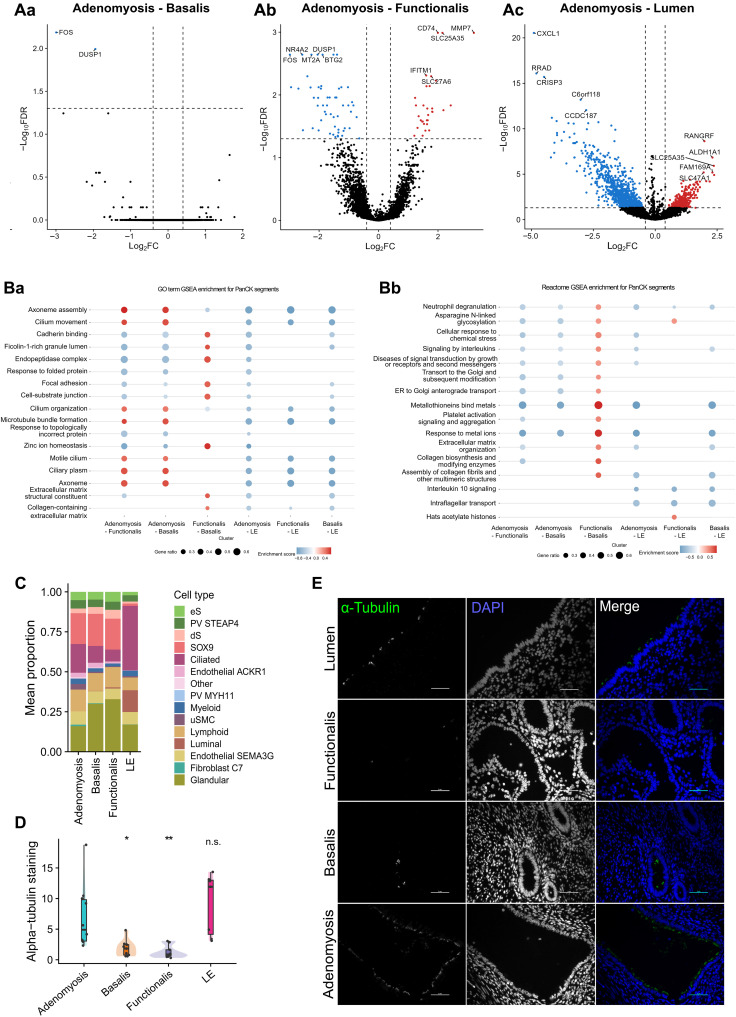
Increased ciliation in adenomyosis lesion epithelium compared to matched eutopic endometrium and luminal epithelium. (**A**) Volcano plots of DEGs between adenomyosis lesion epithelium and basalis (Aa), functionalis (Ab), and luminal epithelium (Ac); *x* axis: log_2_ fold change; *y* axis: −log_10_ FDR-adjusted *P* value. Red: up-regulated genes; blue: down-regulated genes; black: nonsignificant genes. Horizontal line: *P* < 0.05; vertical lines: log_2_ FC thresholds. (**B**) Dot plots of enrichment analysis [(Ba) GO terms and (Bb) Reactome pathways]; dot size: gene ratio; color: enrichment score (red: positive; blue: negative). (**C**) Cell type deconvolution of epithelial cells across adenomyosis lesions and matched basalis, functionalis, and luminal epithelium. (**D**) Boxplots of % α-tubulin–positive epithelial cells in each group; median and range shown. Asterisks *P < 0.05, **P < 0.01, ***P < 0.001; n.s., not significant. (**E**) Representative immunofluorescence staining of α-tubulin (green) and DAPI (blue) in adenomyosis lesions and matched endometrial functionalis and basalis (400×; scale bars, 5 μm).

Adenomyosis lesion epithelium showed fewer significant DEGs compared to matched basalis epithelium than when compared to functionalis or luminal epithelium (Fig. 3, Aa to Ac, and tables S2 to S4). Adenomyosis lesion epithelium was positively enriched for gene sets related to axoneme assembly, cilium movement and organization, and microtubule bundle formation versus both functionalis and basalis epithelium. In contrast, these ciliation-related pathways were negatively enriched in lesion epithelium when compared to luminal epithelium. Gene sets for neutrophil degranulation, interleukin signaling, and extracellular matrix (ECM) organization were negatively enriched in adenomyosis lesion epithelium relative to all eutopic endometrial regions (Fig. 3, Ba and Bb, and table S15). Cell deconvolution and differential abundance analysis suggested a significantly higher proportion of ciliated epithelial cells in adenomyosis lesion epithelium compared to functionalis (*P* = 0.0006) and basalis (*P* = 0.04) but a lower proportion than in luminal epithelium (*P* < 0.0001; Fig. 3C and tables S16 to S18). Immunofluorescence using α-tubulin confirmed these findings: Adenomyosis lesions had a significantly higher percentage of ciliated epithelial cells (median: 4.9%, *n* = 10) than functionalis (0.88%, *n* = 9) and basalis (1.83%, *n* = 9) (*P* < 0.01) but not significantly different from luminal epithelium (11.92%, *n* = 7; Fig. 3, D and E). In addition, the differential gene expression analysis demonstrated up-regulation of cilia-associated genes (*RSPH1*, *RILPL2*, and *TEKT2*) in adenomyosis lesion epithelium relative to eutopic endometrial functionalis and basalis epithelia, consistent with the observed increase in ciliated cell number (fig. S4). Notch signaling pathways were significantly negatively enriched in adenomyosis lesion epithelium compared to eutopic endometrial functionalis and basalis epithelium (fig. S5). Together, these data show that adenomyosis lesion epithelium has an intermediate ciliation phenotype, with more ciliated cells than functionalis and basalis but less than luminal epithelium.

### Adenomyosis lesions exhibit enriched olfactory and mitochondrial pathways

**Fig. 4.**
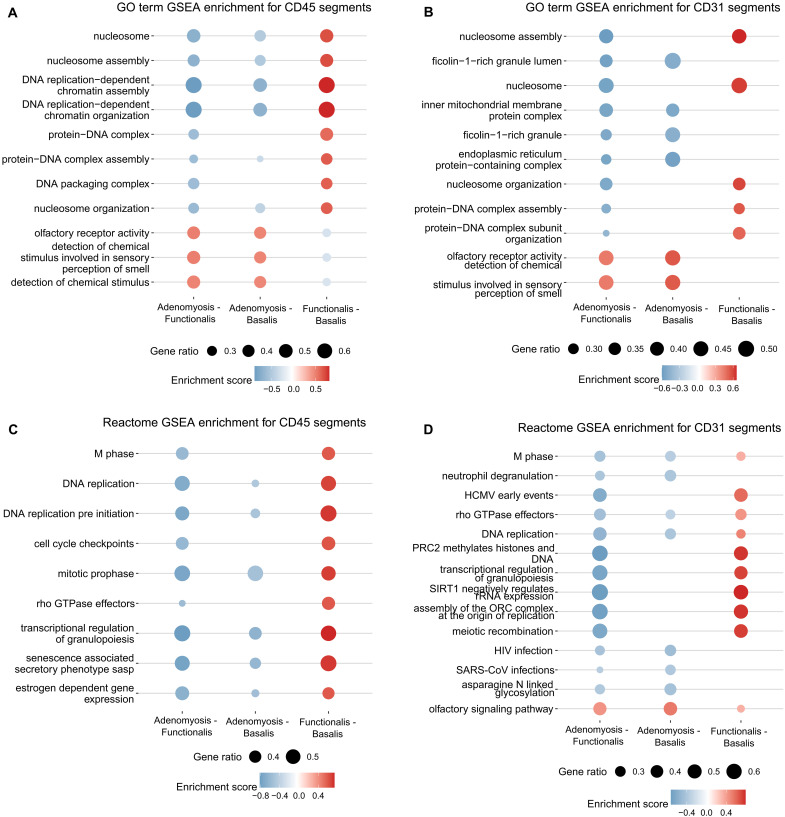
Functional enrichment analysis of DEGs in immune and endothelial cells of adenomyosis lesions compared to matched eutopic endometrial functionalis and basalis. (**A** and **B**) Dot plots of enrichment analysis for DEGs in immune (CD45+) (A) and endothelial (CD31+) (B) cells. Dot size: gene ratio; color: enrichment score (red: positive; blue: negative). (A) GO terms and (**C**) Reactome pathways for immune cell DEGs. (B) GO terms and (**D**) Reactome pathways for endothelial cell DEGs.

Gene sets related to DNA replication, transcription, nucleosome assembly, and protein-DNA complex assembly were negatively enriched in adenomyosis lesion immune cells, indicating reduced proliferation and chromatin remodeling (Fig. 4, A and C, and table S19). Estrogen-dependent gene expression and senescence pathways also showed negative enrichment, suggesting altered hormone responsiveness and cellular aging (Fig. 4C and table S19).

Olfactory signaling pathways were positively enriched in both immune and endothelial cells of adenomyosis lesions compared to endometrium (Fig. 4, A, B, and D, and tables S19 and S20), pointing to potential novel roles in adenomyosis pathophysiology. In adenomyosis lesion endothelial cells, genes involved in mitochondrial function (*MDH2*, *COX7A2*, *COX6B1*, *UQCRC1*, and *SLC25A46*) were significantly down-regulated (tables S9 and S10), and there was negative enrichment of the inner mitochondrial membrane protein complex (Fig. 4B and table S20), suggesting impaired oxidative phosphorylation and adenosine triphosphate (ATP) synthesis.

### Adenomyosis lesions demonstrate an altered immune microenvironment

**Fig. 5.**
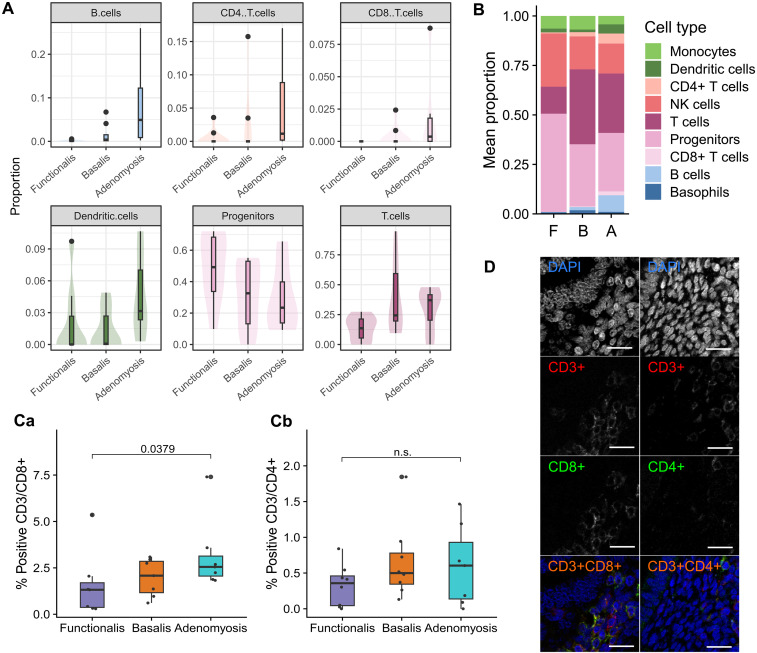
Immune cell composition and T and B cell subsets in adenomyosis lesions compared to matched eutopic endometrial basalis and functionalis. (**A**) Violin plots showing the relative abundance of B cells, CD4+ T cells, CD8+ T cells, dendritic cells, progenitor immune cells, and total T cells in adenomyosis lesions versus basalis versus functionalis. (**B**) Immune cell deconvolution analysis comparing adenomyosis lesions with matched basalis and functionalis immune cells. (**C**) Boxplots of stromal CD3+CD8+ (Ca) and CD3+CD4+ (Cb) T cell percentages in adenomyosis lesion stroma versus eutopic endometrial functionalis stroma versus eutopic endometrial basalis stroma. (**D**) Representative immunofluorescence staining of CD3 (red), CD4/CD8 (green), and DAPI (blue) in adenomyosis lesions (400×; scale bars, 20 μm).

Cell type deconvolution suggested significant changes in immune cell populations within adenomyosis lesions compared to eutopic endometrium (Fig. 5B and tables S21 and S22). In stromal leucocytes, adenomyosis lesions had higher estimated proportions of B cells (*P* < 0.0001), CD8+ T cells (*P* = 0.001), CD4+ T cells (*P* = 0.009), total T cells (*P* = 0.03), and dendritic cells (*P* = 0.01) but fewer progenitor immune cells (*P* = 0.003) than functionalis. Similar increases were seen compared to basalis: more B cells (*P* = 0.0001), CD8+ T cells (*P* = 0.01), CD4+ T cells (*P* = 0.045), total T cells (*P* = 0.03), and dendritic cells (*P* = 0.005).

Immunostaining for T cell markers (CD3, CD4, and CD8; *n* = 7) quantified cytotoxic (CD3+CD8+) and helper (CD3+CD4+) T cells. The percentage of cytotoxic T cells (CD3+CD8+) among all stromal cells was 1.32% (0.29 to 5.35%) in the functionalis, 2.08% (0.61 to 3.09%) in the basalis, and 2.55% (1.83 to 7.4%) in adenomyosis lesions, with significantly more CD3+CD8+ cells in adenomyosis lesions than functionalis (*P* = 0.0379; Fig. 5Ca). Helper T cell percentages were 0.41% (0.03 to 0.84%) in the functionalis, 0.48% (0.13 to 1.85%) in the basalis, and 0.60% (0 to 1.47%) in adenomyosis lesions, with no significant differences observed between regions (Fig. 5Cb). No significant differences were found in intraepithelial leucocyte populations between adenomyosis lesions and either functionalis or basalis (fig. S6).

To address the absence of macrophages in the immune cell deconvolution analysis, CD68 immunohistochemistry (IHC) was performed to detect macrophages in tissue samples. Quantification showed no significant differences in the percentage of stromal CD68+ cells among endometrial functionalis, basalis, or adenomyosis lesions (fig. S7). In contrast, significant increases in CD68+ intraepithelial leucocytes were found in basalis (3.29%, 0.09 to 10.95) versus functionalis (0.81%, 0.09 to 8.0) (*P* < 0.05) and in adenomyosis lesions (3.34%, 0.88 to 6.40) compared with matched functionalis (*P* < 0.05). Overall, the distribution of CD68+ macrophages in lesions was similar to matched basalis tissue (fig. S7).

### Endothelial and immune cells drive the cell-cell communication in adenomyosis lesions

**Fig. 6.**
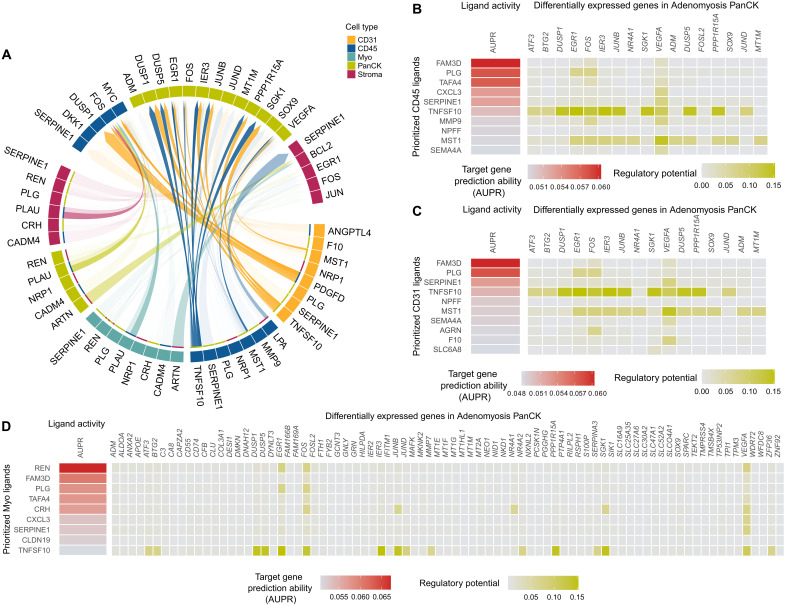
Cell-cell signaling in adenomyosis lesions. (**A**) Circos plot of top-weighted (95th percentile) ligand-receptor pairs; sender genes are those with average counts > 4, and receiver genes are DEGs in ectopic versus eutopic endometrium (adjusted *P* < 0.05, logFC > 1.5 or < −1.5). (**B**) Immune (CD45+)–epithelial (panCK) signaling: AUPR values for prioritized immune ligands and their regulatory potential on epithelial DEGs. (**C**) Endothelial (CD31+)–epithelial (panCK) signaling: AUPR values for endothelial ligands and their regulatory potential on epithelial DEGs. (**D**) Myocyte (panCK−/CD45−/CD31−)–epithelial (panCK+) signaling: AUPR values for myocyte ligands and their regulatory potential on epithelial DEGs.

Having identified unique, cell type–specific transcriptional profiles in adenomyosis lesions, we next examined whether altered intercellular communication contributed to these changes. In contrast to the secretory phase eutopic functionalis, where stromal cells are typically active senders, stromal cells in adenomyosis lesions emerged as the least active sender cell type. Instead, immune and endothelial and myometrial cells were the predominant senders, whereas epithelial cells served as the primary receivers (Fig. 6).

Immune-epithelial signaling involved immune cell *TNFSF10* ligand regulating epithelial genes (*DUSP1*, *DUSP5*, *EGR1*, *FOS*, *IER3*, *JUNB*, *PPP1R15A*, and *SGK1*) via *TNFRSF* receptors (Fig. 6, fig. S8, and tables S23 and S24), likely affecting apoptosis, stress responses, and inflammation in adenomyosis lesion epithelium. Immune cell *SERPINE1* also influenced epithelial *VEGFA* expression through *PLAUR*, *PLAT*, and *PLAU* receptors, supporting angiogenesis and ECM remodeling, whereas *MMP9* from immune cells interacted with epithelial *CD44*, potentially promoting migration and invasion.

Endothelial-immune interactions were mediated by *PDGFD* ligand from endothelial cells acting on immune cell genes (*MYC* and *FOS*) via *PDGFRA* and *PDGFRB* receptors (Fig. 6 and tables S25 and S26). Endothelial-epithelial signaling included *TNFSF10* and *MST1* ligands regulating epithelial genes involved in stress responses (*DUSP1*), transcription (*FOS* and *JUNB*), and angiogenesis (*VEGFA*), suggesting roles in lesion vascularization and epithelial survival (Fig. 6 and tables S27 and S28).

Myometrial *TNFSF10* had strong regulatory effects on genes in adenomyosis lesion epithelial cells linked to stress, inflammation, and cell growth, suggesting roles in ectopic endometrial cell adaptation. Myometrial *CADM4* also showed high regulatory potential on *MYC* and *FOS* in adenomyosis lesion immune cells, potentially supporting their integration via cell adhesion (Fig. 6 and tables S29 to S32).

### In silico identification of adenomyosis lesion-specific drugs

Several compounds with the potential to modulate adenomyosis lesions were identified on the basis of their ability to reverse lesion-specific gene expression profiles. Top-ranked candidates included CGP-55845 and WYE-354 (Table 2), which exhibited modest but consistent inverse correlations (range: −0.2 to −0.3) with the transcriptional signatures of adenomyosis lesions across comparisons with the functionalis and basalis layers of the eutopic endometrium. Consistent drug reversal effects were observed across different cell types within each region, and the top-ranking compounds remained conserved when drug signatures were aggregated by region; full cell type–specific reversal results are presented in tables S33 to S41. Notably, the prioritized compounds are associated with pathways implicated in adenomyosis lesion biology (*29*, *30*), suggesting promising avenues for therapeutic intervention in adenomyosis.

**Table 2.** Summary of potential compounds and mode of action from in silico drug screening of adenomyosis lesions compared to endometrial basalis and functionalis. SARS, severe acute respiratory syndrome; PLK, Polo-like kinase.

Compound	Mode of action
Adenomyosis lesions versus eutopic endometrium	
CGP-55845	GABA receptor antagonist
WYE-354	mTOR inhibitor
Adenomyosis lesions versus endometrial basalis	
WYE-354	mTOR inhibitor
CGP-55845	GABA receptor antagonist
A-443644	AKT inhibitor
Coumarin	Vitamin K antagonist
VU-0420363-1	SARS coronavirus 3C-like protease inhibitor
Adenomyosis lesions versus endometrial functionalis	
Bromhexine	Mucolytic agent
CGP-55845	GABA receptor antagonist
WYE-354	mTOR inhibitor
Dipropyl-5-ct	Serotonin receptor agonist
GR-55562	Serotonin receptor antagonist
HG-6-64-01	RAF inhibitor
GSK-461364	PLK inhibitor

## DISCUSSION

**Fig. 7.**
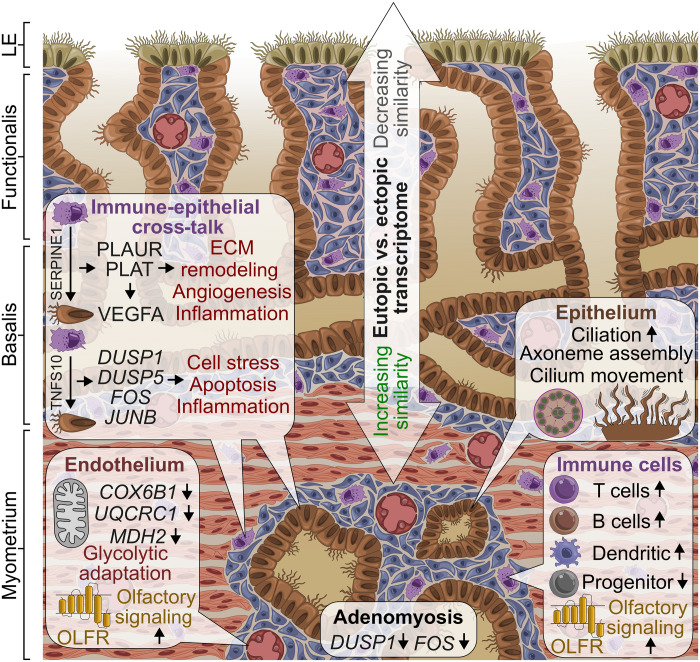
Spatially resolved transcriptional landscape of adenomyosis lesions reveals cell type–specific dysregulation. Schematic representation of full-thickness endometrium with underlying myometrium containing an adenomyosis lesion (not to scale). A gradient of gene expression differences extends from the lesion toward the luminal epithelium (LE), highlighting distinct transcriptional profiles in epithelial, stromal, immune, endothelial, and myometrial compartments. Lesions exhibit increased epithelial ciliation relative to endometrial basalis and functionalis subregions. Enriched pathways include olfactory signaling and mitochondrial pathways. Altered immune microenvironment is characterized by altered immune subpopulations. Immune-epithelial communication is associated with angiogenesis, ECM remodeling, and inflammation. ECM, extracellular matrix; LE, luminal epithelium; OLFR, olfactory receptors.

Adenomyosis is a common and debilitating gynecological disorder. Although existing therapies such as hormonal agents and fertility-sparing surgery can be effective in controlling symptoms, their limited tolerability and acceptability highlight the continuing need for new, mechanism-based treatments for adenomyosis. Progress in understanding its pathogenesis and developing novel treatments has been hindered by limited knowledge into lesion-specific cellular and molecular aberrations. Given its heterogeneity, contextualizing lesions relative to anatomically matched eutopic endometrial regions is essential for identifying therapeutic targets. In this study, we present a large, well-characterized dataset that delineates cell type–resolved and region-resolved transcriptional profile of adenomyosis lesions in comparison to matched full-thickness eutopic endometrium, enabling the identification of lesion-specific molecular abnormalities and potential therapeutic targets. The main findings are summarized in Fig. 7.

Our findings demonstrate that the transcriptome of adenomyosis lesions more closely resembles the endometrial basalis than the functionalis, with greater gene expression divergence observed in comparison with the superficial functionalis endometrium. This observation supports the hypothesis that adenomyosis originates from the invasion of endometrial basalis into the myometrium (*20*, *22*, *31*) and reveals the influence of previously unknown cell types such as myometrial cells on adenomyosis pathogenesis. The interaction between the myometrium and cells within adenomyosis lesions is highlighted by the up-regulation of genes associated with smooth muscle contractility (*ACTG2*, *MYH11*, and *ACTA2*) in adenomyosis lesion stroma compared to the eutopic endometrium. The up-regulation of *ACTA2* and *ACTG2* in these regions may reflect both smooth muscle and myofibroblast activity, indicating potential fibrotic remodeling, as both genes are established markers of myofibroblast differentiation and fibrosis in tissue repair and disease (*30*). Although increased expression of *ACTA2* and *ACTG2* was previously reported in fibroblast clusters of adenomyosis lesions (*32*), our study provides spatial context, implying a direct influence from the surrounding myometrium, supported by cell-cell signaling analysis, which revealed significant cross-talk between the myometrium and adenomyosis lesion epithelium, specifically showing that myometrial *TNFSF10* and *CADM4* exerted strong regulatory influences over lesion epithelial and immune cells, respectively. This proposes a regulatory potential for the *TNFSF10* ligand on genes within adenomyosis lesion epithelial cells, including *DUSP1* and *DUSP5*. *DUSP1* was down-regulated in epithelial, stromal, and immune cells of adenomyosis lesions compared to eutopic endometrium. *DUSP1* negatively regulates members of the mitogen-activated protein kinase (MAPK) superfamily (*33*), which have functions in cellular proliferation and differentiation (*34*). This down-regulation could lead to increased MAPK activity, potentially promoting the abnormal cell proliferation observed in adenomyosis.

Ciliated cells are most abundant in the endometrial luminal epithelium, located farthest from the adenomyotic lesions. Ciliated epithelial cells in the endometrium exhibit cyclical variation, increasing during the 17β-estradiol (E_2_)–driven proliferative phase (*35*, *36*). However, the existing data on ciliated cells in adenomyosis lesions are limited. Yildiz *et al.* (*32*) reported 3% of epithelial cells were ciliated in eutopic endometrium and 1% in adenomyosis lesions. As single-cell isolation removes spatial context, the subregional origin of these cells remains unclear. Notably, a recent integrative spatial and single-cell transcriptomic study demonstrated that ectopic endometrial glands in adenomyosis are enriched with ciliated epithelial cells (*37*). The increased presence of ciliated epithelial cells in adenomyosis lesions may result from local hormonal dysregulation. Ciliogenesis is induced by E_2_ exposure (*38*), and elevated local E_2_ production in adenomyosis lesions (*18*) could drive this process. Single-cell transcriptional profiling of epithelial abnormalities in ovarian endometriosis found an increased proportion of ciliated cells in the ectopic endometrium compared to the matched eutopic endometrium (*39*). Furthermore, down-regulation of *SULT1E1* was observed in ciliated epithelial cells in ectopic lesions, indicating a higher estradiol level. The healthy endometrium regenerates from the stem/progenitor cells located in the endometrial basalis and grows upward toward the lumen (*15*). Adenomyosis lesions may arise from misdirected growth of stem/progenitor cells in the basalis into the myometrium (*21*, *40*). The transcriptome of lesion epithelial cells more closely resembles that of the basalis than the luminal epithelium, suggesting environmental influences due to proximity to the myometrium. Consequently, we propose that the epithelial cells within adenomyosis lesions are derived from the same stem/progenitor cells that give rise to endometrial regeneration. However, these cells are subject to environmental influences due to their location, resulting in a gene expression profile that more closely resembles that of the endometrial basalis rather than the luminal epithelium. Disruption of the junctional zone may alter signaling cues from the subendometrial myometrium that normally guide basalis epithelial progenitors, providing a potential link between the basalis-derived origin and collective cell migration hypotheses (*41*, *42*). Negative enrichment of notch signaling pathways in adenomyosis lesions, compared to the eutopic endometrium, coincides with the increased ciliation phenotype in lesion epithelium. This relationship is supported by evidence that attenuation of Notch signaling promotes ciliated cell differentiation (*43*–*46*), highlighting a direct mechanistic link between reduced Notch activity and enhances epithelial ciliogenesis in adenomyosis lesions. The observed *CADM4* mediated myometrial-immune cell cross-talk could affect cilia function as it is implicated in primary ciliary dyskinesia. Abnormalities in microtubule formation have been reported in adenomyosis (*47*), and dysfunctional ciliated epithelial cells are implicated in infertility and recurrent pregnancy loss (*48*, *49*). Further research into their role in adenomyosis pathogenesis is warranted.

Up-regulation of genes related to mitogenesis (*FOSb*, *FOS*, and *JUN*) have been identified in the eutopic endometrium of women with adenomyosis (*32*, *50*, *51*). Our data corroborated these findings, demonstrating up-regulation of *FOS* and *JUN* in the eutopic endometrium compared to adenomyosis lesions, specific to epithelial, stromal, and immune cell populations. The FOS and JUN family proteins form the AP-1 transcription complex, regulating cell proliferation, differentiation, apoptosis, and stress response (*52*, *53*). The down-regulation of *FOS* and *JUNB* in adenomyosis lesions may contribute to its pathogenesis through aberrant molecular processes (*54*–*56*). We uncovered cell type–specific pathophysiological processes in adenomyosis lesions, which highlights the importance of studying adenomyosis lesions with spatially resolved analysis allowing consideration of how different cell types within adenomyosis lesions contribute to the disease. Olfactory signaling pathways were enriched in lesion immune and endothelial cells. Although olfactory receptors (ORs) are traditionally associated with nasal epithelium, they are ectopically expressed in the endometrium (*57*). If adenomyosis lesions constitute a hypoxic microenvironment, as suggested by overexpression of hypoxia inducible factor-1α (HIF1α) and vascular endothelial growth factor (VEGF) in adenomyosis lesions, this could elevate insulin-like growth factor 1 (IGF-1) expression (*58*) and may be linked to olfactory signaling pathways. Specifically, OR5H2 has been shown to regulate endometrial cancer cell proliferation via IGF-1 signaling (*59*), raising the possibility that similar mechanisms contribute to adenomyosis lesion pathogenesis. Further investigation into OR-mediated pathways in adenomyosis is warranted.

Endothelial cells in adenomyosis lesions exhibit down-regulation of mitochondrial genes relative to eutopic endometrium, suggesting a metabolic shift. Mitochondrial dysfunction can impair ATP production and affect cell survival and function, and previous studies have reported dysregulated mitochondrial pathways (*60*) and decreased *TOMM20* expression (*61*) in adenomyosis endometrium. Our spatially resolved data indicate that this dysfunction is localized to the lesion endothelium and raised the possibility that adenomyosis lesions may adopt a Warburg-like reliance on glycolysis to meet energy demands (*62*). Given glycolysis-driven growth in endometriosis (*63*), targeting these metabolic alterations may represent a therapeutic strategy for adenomyosis.

Adenomyosis lesions exhibit distinct endothelial gene expression profiles compared to endometrial basalis and functionalis, reflecting differences in myometrial versus endometrial vasculature. These differences likely indicate altered angiogenic and vascular remodeling processes within lesions, consistent with increased microvessel density and vascular endothelial growth factor (*VEGF*) expression reported in adenomyosis (*58*, *64*). Such vascular abnormalities may contribute to heavy menstrual bleeding through increased vascular permeability and dysregulated blood flow. Further studies comparing lesion vasculature with normal endometrium are needed to clarify these mechanisms.

Adenomyosis lesions exhibit a shift toward adaptive immunity and chronic inflammation, aligning with previous reports of inflammation in adenomyosis (*8*, *65*), evidenced by increased of B cells, T cells (particularly cytotoxic subsets), and dendritic cells in lesions, which suggests a sustained inflammatory environment that may play a role in lesion survival (*66*). Gene expression profiles reveal up-regulation of T cell–related genes and down-regulation of proliferation-associated genes, indicative of terminal differentiation of immune cells. This is consistent with a previous study demonstrating elevated T cell presence in adenomyosis lesions (*67*). The concomitant negative enrichment of DNA replication, transcription, and nucleosome assembly pathways further supports a more differentiated immune state that may perpetuate inflammation and impair tissue repair. Although immune cell deconvolution may under detect macrophages due to limitations in reference data, CD68 IHC confirmed their presence and increased intraepithelial localization in adenomyosis lesions and basalis compared to functionalis, supporting a role for macrophages in adenomyosis pathology. Consistent with the recent spatial and single-cell transcriptomic study (*37*), immune-vascular cross-talk may also contribute to adenomyosis pathogenesis. That study reported enrichment of CD4+ and LYVE1+ macrophages in untreated adenomyosis, together with a proangiogenic microenvironment that was partially attenuated following GnRHα treatment, suggesting potential immunomodulatory and antiangiogenic effects of GnRHα. Although macrophage phenotypes and angiogenic markers were not directly assessed by IHC in the present study, our data provide spatial context for these observations by demonstrating region-specific transcriptional differences across the endometrium and adenomyotic lesions. Differences in menstrual cycle phase, tissue sampling strategy, and cohort composition between the two studies may account for the divergent immune and vascular signatures observed. Cell-cell communication analyses implicate TNF Superfamily Member 10 (TNFSF10)-mediated immune-epithelial cross-talk, which may drive epithelial-mesenchymal transition (EMT) via MAPK8/JNK (c-Jun N-terminal kinase) signaling (*68*). TNFSF10 may influence the local immune environment to indirectly promote EMT; hypoxia-induced modulation of TNFSF10 expression has been implicated in EMT in proximal tubular epithelial cells (*69*). Furthermore, TNFSF10’s ability to induce selective apoptosis in epithelial cells via death receptor signaling (*70*) may create a microenvironment conducive to EMT by eliminating nonmesenchymal cells and enabling mesenchymal phenotype acquisition.

Adenomyosis lesions demonstrate *SERPINE1*-mediated immune-epithelial signaling through *VEGFA*, which suggests promotion of neovascularization and involvement in fibrosis and ECM remodeling. *SERPINE1* encodes plasminogen activator-1 (PAI-1), a serine protease inhibitor regulating fibrinolysis, ECM remodeling, and inflammation (*71*). Elevated *PAI-1* expression in adenomyosis lesions positively correlates with dysmenorrhea severity and lesion fibrosis (*72*), whereas concomitant VEGFA up-regulation promotes angiogenesis. Together, VEGFA and PAI-1 may perpetuate vascularized fibrotic lesions, contributing to pain in adenomyosis. Such pathological cross-talk may necessitate combined targeting of fibrinolytic and angiogenesis pathways in adenomyosis management.

In silico drug repositioning identified several compounds with the potential to selectively target adenomyosis lesions while sparing the correctly located eutopic endometrium. This is important given that current treatments, primarily hormone therapies or hysterectomy, largely address symptoms rather than the underlying disease. Compounds such as CGP-55845 and WYE-354 were prioritized for their ability to exploit molecular features unique to adenomyosis. Although direct evidence for their effects in gynecological diseases is limited, their mechanisms are well characterized; CGP-55845 is a γ-aminobutyric acid type A (GABA_B_) receptor antagonist linked to cell growth and migration (*73*), and WYE-354 is a selective mechanistic target of rapamycin (mTOR) inhibitor with demonstrated antiproliferative and pro-apoptotic effects in cancer models (*74*). WYE-354 is particularly promising given the central role of the PI3K/Akt/mTOR pathway in the pathophysiology of endometriosis (*29*, *75*) and potentially adenomyosis (*30*). mTOR signaling has been observed in both bulk transcriptomic (*60*) and single-cell analyses (*61*) of the endometrium from women with adenomyosis. mTOR activation promotes estrogen receptor expression (*76*), stromal cell–derived factor 1 secretion, and angiogenesis, processes that support ectopic endometrial lesions (*77*). Preclinical studies suggest that mTOR inhibition can reduce stromal cell proliferation, migration, and survival (*78*) while promoting autophagy and modulating immune responses (*78*). mTOR activation promotes estrogen receptor expression. These findings support mTOR as a therapeutic target in adenomyosis and underscore WYE-354’s potential to modify disease by addressing lesion-specific molecular changes.

This study has several limitations that should be considered. First, all samples were collected during the secretory phase of the menstrual cycle, selected for its marked phenotypic variability between endometrial subregions; as such, our findings may not be generalizable to other menstrual phases. Although the cohort included samples spanning the early, mid, and late secretory subphases, subanalyses revealed no significant impact of subphase variation on gene expression or cellular composition. Consequently, the results should be interpreted as representative of the secretory phase as a whole rather than specific subphases. Although there is growing recognition of distinct adenomyosis subtypes based on lesion location [e.g., diffuse versus focal and intrinsic versus extrinsic (*79*)], there is a lack of standardized diagnostic criteria. In our study, all adenomyosis lesions were confined to the inner myometrium, consistent with intrinsic adenomyosis, which aligns with the demographic profile of the cohort (multiparous women aged 40 to 50 years). However, our study did not permit investigation of potential differences in molecular or histological features across phenotypes, which should be explored in future studies. Adenomyosis lesions may vary in developmental stage and fibrotic remodeling, even within the same uterus, which could influence compartmental analyses; however, as all participants had intrinsic-type adenomyosis, such variability was accounted for as far as possible in the analysis. Our study compares adenomyosis lesions with matched eutopic endometrium, thus our conclusions are specific to this comparison and cannot be generalized to healthy endometrium. The coexistence of multiple chronic gynecological pathologies, such as fibroids and endometriosis, is common in hysterectomy specimens, making it rare to obtain truly normal, hormone-naïve uterine tissue. This limitation is inherent to studies involving surgical specimens and should be considered when interpreting molecular and cellular differences reported in adenomyosis. Progesterone resistance and reduced progesterone receptor (PGR) expression in endometrial stromal cells are established features in adenomyosis, as well as in related conditions such as endometriosis and fibroids (*80*–*82*). Although our study did not identify differential regulation of progesterone signaling genes between lesions and matched eutopic endometrium, likely due to the within-patient design, it is important to recognize that molecular mechanisms underpinning progesterone resistance remain incompletely understood. Disease-control comparisons will be necessary to directly address global endometrial progesterone responsiveness in future research. Despite these limitations, our comprehensive spatial transcriptomic profiling of adenomyosis lesions and matched eutopic endometrium provides valuable insights into disease pathogenesis and highlights potential therapeutic targets. To translate these findings into clinical applications, future studies should incorporate larger, well-characterized cohorts stratified by menstrual phase and adenomyosis subtype, with detailed region-specific analyses of the eutopic endometrium. Collaborative efforts will be essential to validate these results, further delineate immunological and molecular heterogeneity in adenomyosis, and advance the development of targeted, lesion-specific therapies.

## MATERIALS AND METHODS

### Experimental design

**Fig. 8.**
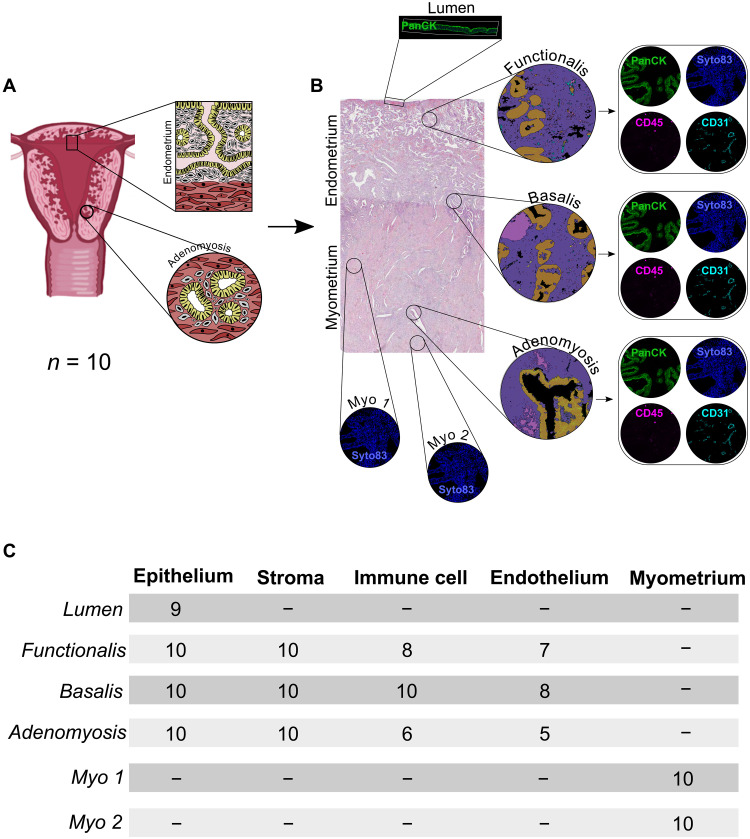
Endomyometrial regions and cell types analyzed. (**A**) Biopsies were obtained from eutopic endometrium and matched adenomyosis lesions from each included patient (*n* = 10). (**B**) ROIs and cell type identification using immunofluorescence markers: panCK+, epithelial; Syto83+/panCK−/CD45−/CD31−, stromal; CD45+, immune cell; CD31+, endothelium; and Syto83+/panCK−/CD45−/CD31−, myometrium. Two myometrial regions were selected: Myo 1, close to the junctional zone, and Myo 2, close to the adenomyosis lesion. (**C**) Summary of regions and cell types used for transcriptional analysis.

The objective of this study was to elucidate the spatial transcriptomic profile of adenomyosis lesions with matched eutopic endometrial subregions, with a focus on specific cell types and tissue compartments. Full-thickness endometrial biopsies and matched myometrium containing adenomyosis lesions were prospectively collected from ten premenopausal women undergoing hysterectomy for pelvic pain and/or heavy menstrual bleeding. Prespecified regions of interest (ROIs) were identified on digital histological images, including the luminal epithelium, endometrial functionalis and basalis, adenomyosis lesions, and two distinct myometrial locations (Fig. 8, fig. S9, and fig. S10). Spatial transcriptomics was optimized for each tissue type to enable high-resolution gene expression analysis. The experimental design was intrapatient and comparative, using endometrium, adenomyosis lesions, and myometrium from the same individuals to control interindividual variability and to mitigate confounding effects of comorbid conditions. This approach ensured that observed transcriptional differences reflected true region-specific molecular alterations rather than between-subject variation. The study was designed to identify DEGs, functionally enriched pathways, and cell-cell communication networks within adenomyosis lesions compared to matched endometrial subregions and to nominate potential drug targets for future therapeutic development.

### Human tissues

Collection and use of all samples were approved by the Liverpool Adult Research Ethics Committee (REC references: 09/H1005/55 and 11/H1005/4), with informed written consent obtained from all participants. Full-thickness endometrial biopsies and matched myometrium containing adenomyosis lesions were collected from 10 premenopausal patients undergoing hysterectomy for pelvic pain and/or heavy menstrual bleeding. An experienced gynecological pathologist confirmed adenomyosis diagnosis and determined the menstrual cycle stage of eutopic endometrium using Noyes criteria (*83*, *84*) and patient-reported last menstrual period. None of the patients had used hormonal treatments in the previous 3 months. Adenomyosis subtype was identified by histopathological examination of the resected uterus. Adenomyosis lesions were confirmed by hematoxylin and eosin staining, defined as endometrial glands and stroma >2.5 mm below the endomyometrial junction. Demographics were summarized using means/medians for continuous data and counts/percentages for categorical data.

### NanoString GeoMx digital spatial profiling

Formalin-fixed paraffin-embedded (FFPE) tissue sections (5 μm in diameter) were mounted onto slides (Thermo Fisher Scientific Superfrost Plus) and prepared according to the NanoString RNA Slide preparation protocol (*85*). Sections were immunostained with fluorophore-labeled antibodies (Table 2) and nuclear marker SYTO83 (0.4 nM, Thermo Fisher Scientific) and treated with an autofluorescence quenching kit (Vector Labs, SP-8400). Slides were scanned immediately or stored at 4°C for up to 7 days.

ROIs were selected from digital images, including luminal epithelium, endometrial functionalis and basalis, adenomyosis lesions, and two myometrial locations. Luminal epithelium was defined as the single layer of columnar epithelial cells lining the endometrial cavity. The endometrial functionalis and basalis layers were distinguished histologically: Functionalis features tortuous glands with a single epithelial layer surrounded by edematous stroma, whereas basalis shows compact stroma with narrower, pseudostratified glands exhibiting horizontal branching, consistent with established histological descriptions (*15*). Myometrial regions were collected within 400 μm of either the endometrial-myometrial junction (Myo1) or the nearest adenomyosis lesion (Myo2) to ensure spatial proximity consistency across samples and did not include fibroid tissue. Endometrial cell types were identified using marker expression: epithelial cells (panCK+), leucocytes (CD45+), and endothelial cells (CD31+). Endometrial stromal cells were defined as panCK−CD45−/CD31− and localized specifically within the endometrium. Myometrial cells, although sharing the same marker-negative profile (panCK−/CD45−/CD31−), were distinguished by their spatial location within anatomically defined myometrium. Comparative analyses were performed between epithelial cells from the luminal epithelium, functionalis glandular epithelium, basalis glandular epithelium, and adenomyosis lesion epithelium, and between stromal cells, leucocytes, and endothelial cells from the functionalis, basalis, and adenomyosis lesion regions. Figure 8 illustrates the endomyometrial regions and cell types selected for comparison.

### Library preparation and RNA-seq

Photocleaved oligonucleotides from ROI aspirates, containing target-specific readout tags and unique molecular identifiers (UMIs), were used for library preparation. Libraries were quality checked using a Bioanalyzer system (Agilent) and quantified with a Qubit high-sensitivity DNA kit. Sequencing was conducted on an Illumina NovaSeq system using NovaSeq S1 flow cells with paired-end 2 x 50–base pair (bp) reads, generating ~1300 million clusters per cell. Digital count conversion (DCC) files were produced for each target probe.

### Spatial data analysis

Data analysis was performed in R version 4.3.2 (R Core Team, 2023), using the NanoString-developed packages NanoStringNCTools (*86*), GeomxTools (*87*), and GeoDiff (*88*). Unless stated otherwise, visualizations were produced using ggplot2 and *P* values were adjusted using the Benjamini-Hochberg method [false discovery rate (FDR) < 0.05]. Code for data analysis was generated and adapted from a preliminary version of the analysis pipeline (*89*, *90*).

As a quality check, we confirmed that key marker genes exhibited expected expression patterns within relevant cell types (fig. S11A). In addition, we generated “mock bulk” RNA-seq data by averaging expression across all segments and comparing functionalis and basalis tissues, identifying DEGs consistent with known regional and functional biology (fig. S11B).

Sample-level quality control was conducted using NanoStringNCTools to remove poorly performing ROIs, default parameters were used, except for minimum cell count, which was set to 20. Probe-level quality control and normalization were performed using the GeoDiff package. A Poisson background model (with slide as a grouping variable) and background score test were used to determine which probes were outliers (i.e., those with counts significantly lower or higher than expected under a Poisson distribution) and to determine which targets were expressed above the background of the negative probes. Size factors were estimated using a negative binomial threshold model, and the counts were subsequently normalized using a Poisson threshold model-based approach, followed by log_2_ transformation.

Principal components analysis (PCA) and Eigencor plot generation were performed using PCATools (*91*). t-distributed stochastic neighbor embedding (t-SNE) plots, along with PCA, were generated to visualize sample relationships and clustering patterns (figs. S12 and S13). Additional data exploration pairwise Pearson correlation analysis between samples. Hierarchical clustering of the resulting correlation coefficients was performed using the “ward D2” algorithm, with results visualized using pheatmap (*92*) (fig. S14).

Differential gene expression analysis was carried out on the normalized data using the limma package, with limma’s duplicateCorrelation method used to account for repeated measures from the same patient and the limma-trend pipeline to model the data (*93*, *94*). Gene Set Enrichment Analysis (GSEA) was conducted using the fgsea algorithm, implemented through ClusterProfiler’s compareClusters function (*95*), using Reactome pathways (*96*) from the MSigDB (*97*) and Gene Ontology (GO) terms. Ligand activity prediction used NicheNet (*98*). Genes with average counts greater than 4 across all segments of a given cell type were considered expressed in the sender cells. In the receiver cells, genes of interest were those differentially expressed between ectopic and eutopic endometrium and with a higher log fold change (logFC; adjusted *P* value < 0.05 and logFC > 1.5 or < −1.5), focusing on the strongest signals likely to contribute to adenomyosis pathogenesis. The area under the precision-recall curve (AUPR) was used to identify the top ligands, and ligand-target link weights for these ligands were subsequently inferred using the prior knowledge table.

To identify potential drug repositioning candidates for adenomyosis, we used a transcriptional signature–based drug repurposing approach, comparing adenomyosis gene expression profiles with perturbational signatures from the Library of Integrated Network-based Cellular Signatures (LINCS) database. Transcriptomic similarity was assessed using the signatureSearch package, based on differential expression results. The “Cor” method was used to calculate Pearson correlation scores between log fold change values of disease signatures and LINCS drug profiles. As a complementary approach, the LINCS method was also used, where the top 300 up-regulated and down-regulated genes (FDR ≤ 0.05) were used to construct query signatures. All similarity calculations were run using the gess_cor() or gess_lincs() functions, referencing the LINCS database (EH3226) accessed via the ExperimentHub. Compounds were filtered on the basis of mechanism of action (MoA) annotations from the Touchstone dataset. Drugs negatively correlated (Pearson *r* < −0.2) with adenomyosis signatures were flagged as candidates for further exploration. In addition, we performed Drug Set Enrichment Analysis (DSEA) using the calculateDSEA() function to identify enriched pathways and target classes among prioritized compounds. Transcriptome reversal scores were computed for each cell type using the Pearson correlation coefficient (cor_score) between the drug-induced and lesion-derived expression signatures. Cell type–specific signatures were also aggregated by lesion region (adenomyotic lesions and eutopic endometrium) to identify drugs conserved across regions.

### Comparison with existing single-ell data

Endometrial single-cell sequencing data from the Reproductive Cell Atlas (*24*) was downloaded from https://cellgeni.cog.sanger.ac.uk/vento/reproductivecellatlas/endometrium_all.h5ad and converted to a Seurat (v 5.0.3) (*99*) data object with SeuratDisk (v 0.0.0.9021). Immune cells were annotated using SingleR (*100*) using the Monaco Immune Cell reference (GSE107011) (*101*). The provided broad and fine cell type annotations, in addition to custom immune annotations, were used to create custom profile matrices with SpatialDecon (v 1.12.3) (*102*) and subsequently used to estimate the proportion of cell types in each segment of the GeoMx spatial transcriptomics data with default settings. Differential cell abundance analysis was carried out on the estimated proportions using the propellor method from Speckle (*103*).

### Immunostaining

FFPE tissue sections (3 μm) were deparaffinized, rehydrated, and underwent heat-induced antigen retrieval (HIAR) in citrate buffer (pH 6.0). Endogenous peroxidase activity was quenched by 0.3% H_2_O_2_. Primary antibodies (table S42) were applied. For IHC, antigen detection used the ImmPRESS HRP IgG polymer system (Vector Labs) and ImmPACT DAB substrate kit for visualization. Sections were counterstained with Gill 2 hematoxylin, dehydrated, and mounted with Consul-Mount. For immunofluorescence, sections were incubated with fluorophore-conjugated secondary antibodies (goat anti-mouse IgG Alexa Fluor 488 and anti-rabbit IgG Alexa Fluor 555, Invitrogen, 1:1000) for 2 hours at room temperature. Slides were mounted with VECTASHIELD HardSet Antifade Mounting Medium with 4′,6-diamidino-2-phenylindole (DAPI) (Vector Labs, SP-8500).

### Statistical analysis of immunostaining

Immunofluorescence staining was quantified across multiple regions per sample, including adenomyosis lesions, endometrial basalis, and functionalis, and, for α-tubulin, luminal epithelium. For each region, five randomly selected images were acquired per sample. All image analyses were performed using ImageJ (NIH) in conjunction with the StarDist plug-in to automatically quantify total nuclei and distinguish stromal from epithelial compartments. α-Tubulin–positive cells, or dual-positive cells (CD3+CD4+ or CD3+CD8+), were manually counted in ImageJ, and their proportions were calculated relative to the total number of cells per image. To ensure reproducibility, a subset of images was independently analyzed by multiple observers. Statistical analyses were conducted using nonparametric tests, as appropriate for data distribution. Paired comparisons were assessed with the Wilcoxon signed-rank test, whereas unpaired comparisons were evaluated with the Mann-Whitney *U* test.
