# Supplementary material for: Spatial transcriptomics uncovers the hybrid molecular identity, ciliated phenotype, and immune signature of adenomyosis lesions
Source: Sci Adv. 2026 Jun 24;12(26):eaea6379. doi: 10.1126/sciadv.aea6379 (PMC13292934; doi:10.1126/sciadv.aea6379)
Supplement: Supplementary file 1 — Figs. S1 to S14 Tables S1 to S32, and S42 Legends for tables S33 to S41 [file sciadv.aea6379_sm.pdf]

Science Advances
AAAS

Supplementary Materials for

Spatial transcriptomics uncovers the hybrid molecular identity, ciliated phenotype, and immune signature of adenomyosis lesions

Alison Maclean *et al.*

Corresponding author: Alison Maclean, amaclean@liverpool.ac.uk

Sci. Adv. **12**, eaea6379 (2026)
DOI: 10.1126/sciadv.aea6379

The PDF file includes:

Figs. S1 to S14
Tables S1 to S32, and S42
Legends for tables S33 to S41

Other Supplementary Material for this manuscript includes the following:

Tables S33 to S41

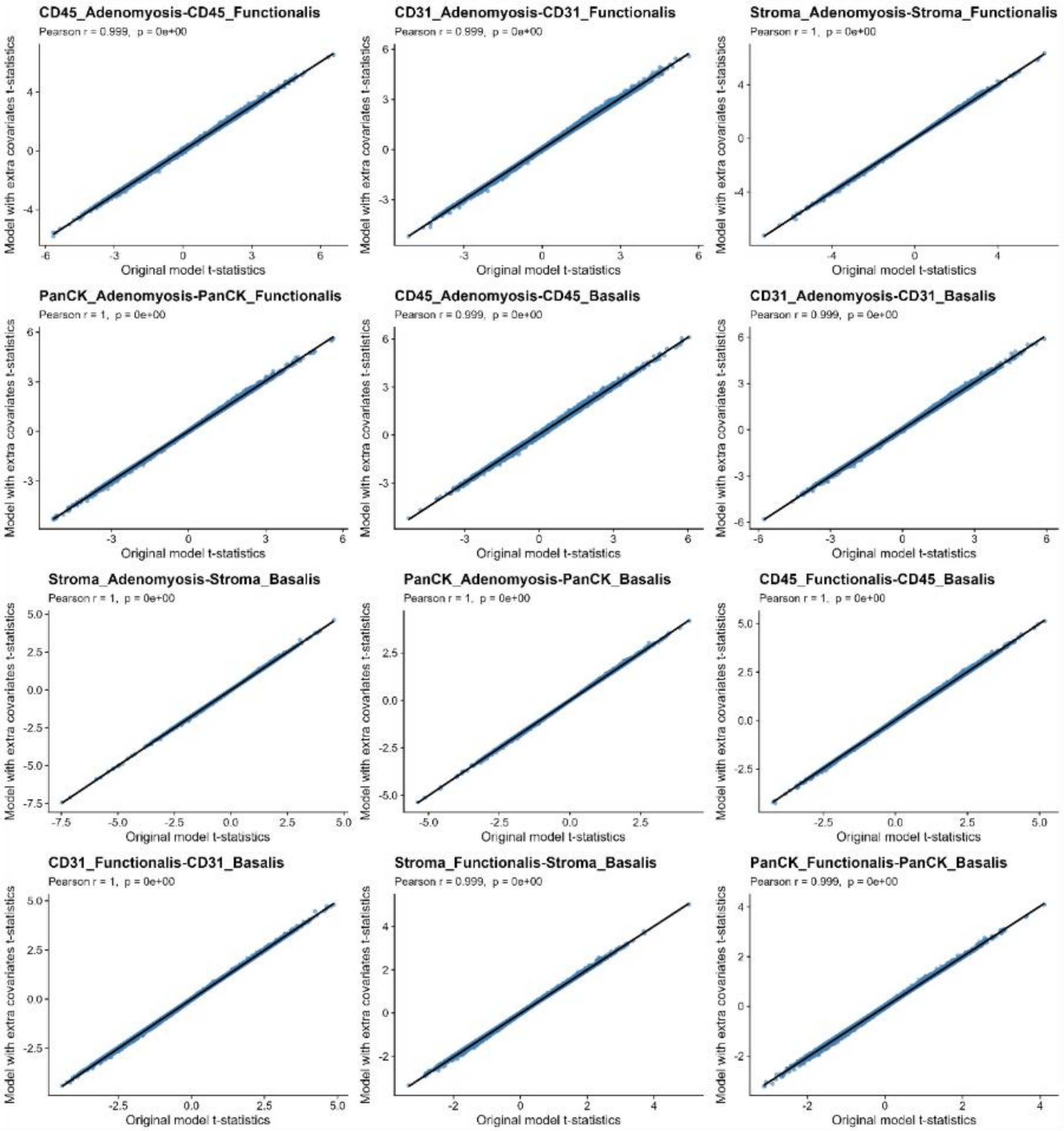

Figure S1: Sensitivity analysis to demonstrate robustness of findings to comorbidity status and inclusion of covariates. Scatter plots comparing t-statistics from original differential expression model (x-axis) versus model adjusted for secretory sub-phase, fibroid and endometriosis status (y-axis) for 12 primary comparisons. Pearson correlation coefficients (r) and p-values shown. Near-perfect correlation (r = 0.996–1.0) across all contrasts demonstrates that results are not confounded by presence of fibroids or endometriosis or the sub-phase of the cycle. Each point represents one gene; blue points indicate statistically significant genes (FDR < 0.05) in at least one model.

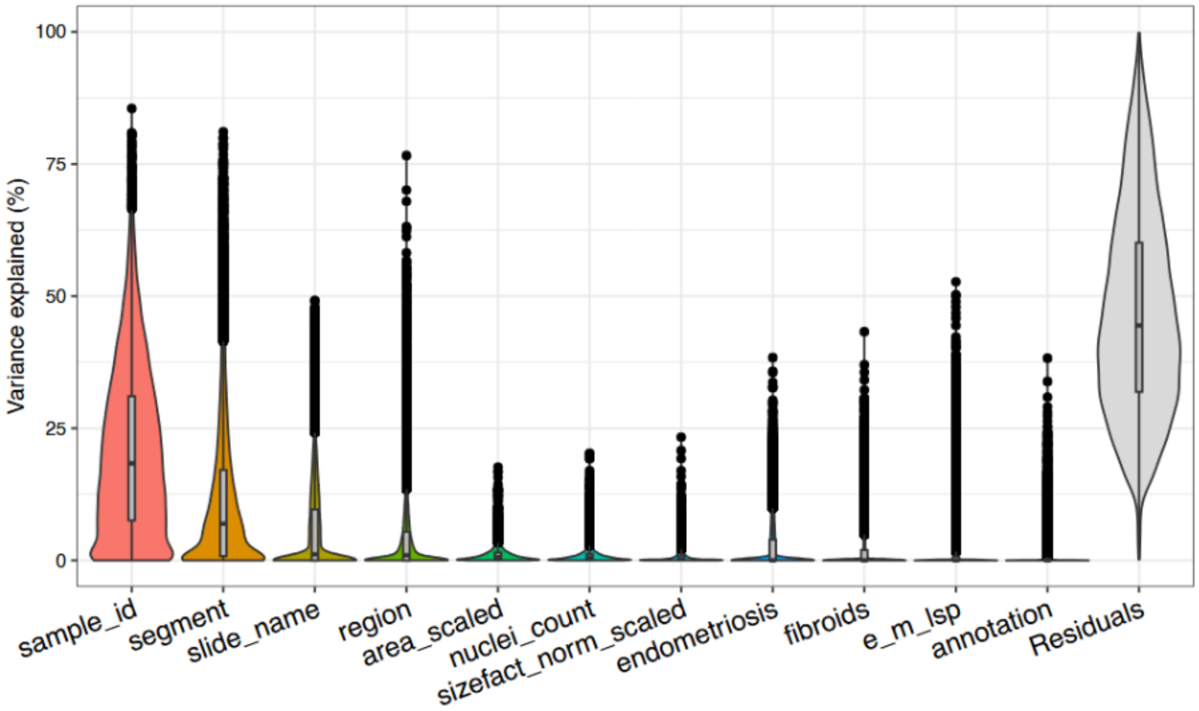

Figure S2: Variance partitioning analysis demonstrates patient-level factors dominate gene expression variation. Violin plots describe relative explained variance by the experimental variables. Variables are sorted sequentially by highest median of explained variance to lowest.

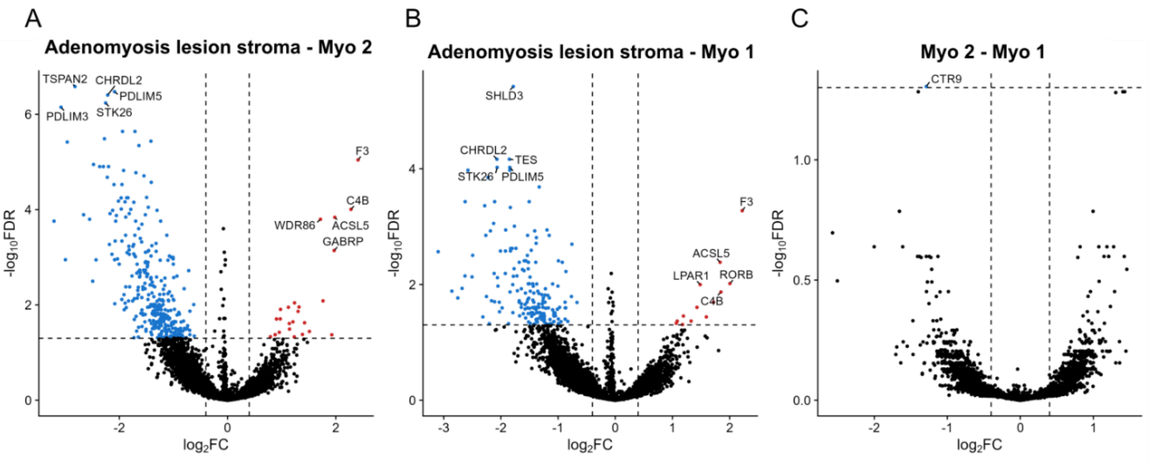

Figure S3. Volcano plots of differentially expressed genes between adenomyosis lesion stroma and myometrial regions Myo 1 (within 400 µm of the endometrial–myometrial junction) and Myo 2 (within 400 µm of the nearest adenomyosis lesion). A: Adenomyosis lesion stroma vs. Myo 2. B: Adenomyosis lesion stroma vs. Myo 1. C: Myo 2 vs. Myo 1. X-axis: log2 fold change; y-axis: –log10 FDR-adjusted p-value. Red: upregulated; blue: downregulated; black: non-significant genes. Horizontal line: $p < 0.05$; vertical lines: log2 FC thresholds.

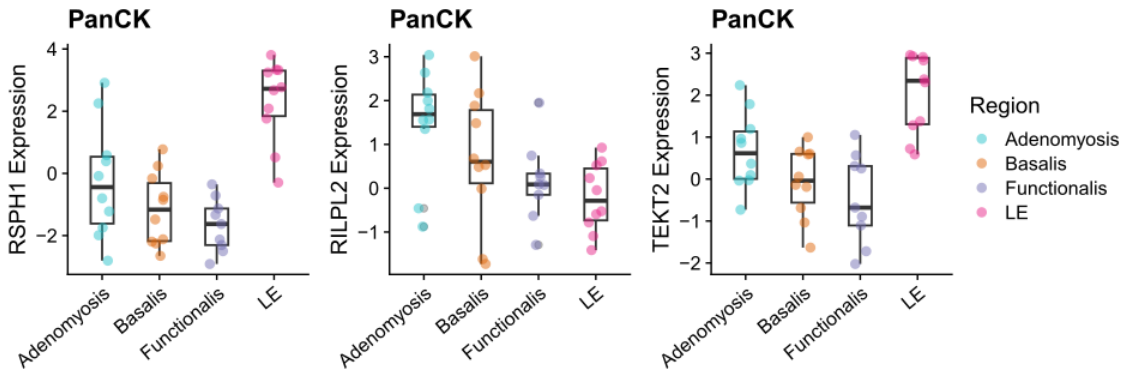

Figure S4. Boxplots of cilia-associated gene expression (*RSPH1, RILPL2, TEKT2)* in adenomyosis lesion epithelium, eutopic endometrial functionalis epithelium, eutopic endometrial basalis epithelium, and luminal epithelium; median and range shown. LE, luminal epithelium.

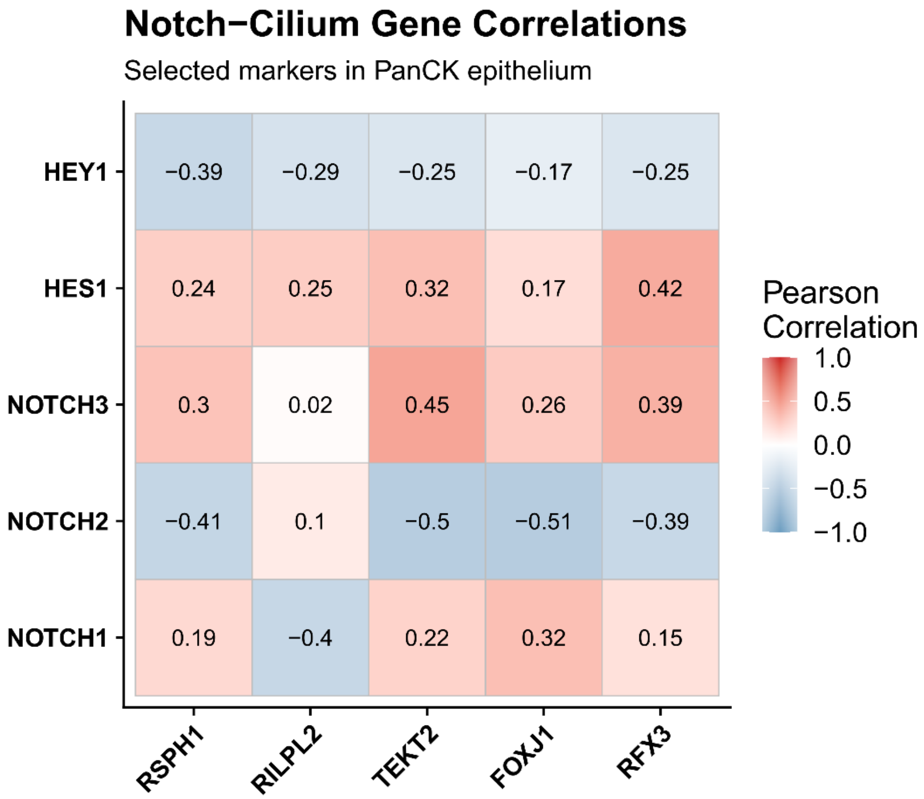

Figure S5. Pearson's correlation of normalized expression levels of key Notch signaling genes with genes relating to ciliation across the epithelial (PanCK) samples.

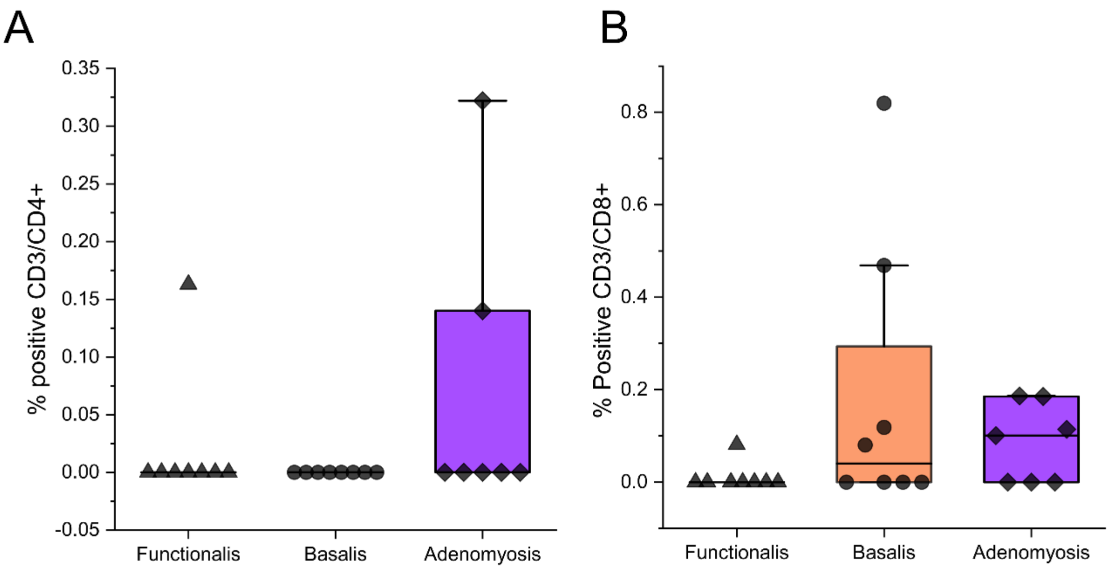

	CD3+CD4+		
	Median	Range	P value
F	0	0 - 0.163	NS
B	0	0 - 0	NS
A	0	0.148 - 0.323	NS

	CD3+CD8+		
	Median	Range	P value
F	0	0 - 0.081	NS
B	0.04	0 - 0.469	NS
A	0.101	0 -0.186	NS

Figure S6. Intraepithelial leucocyte (IEL) composition in adenomyosis lesions compared to matched eutopic endometrial basalis and functionalis. A: Boxplot and tabulated summary of percentage of CD3+CD4+ T cells, and B: Boxplot and tabulated summary of percentage positive CD3+CD8+ T cells in adenomyosis lesions versus matched eutopic endometrium. The middle line represents the median value and whiskers denote minimum and maximum values. F, functionalis; B, basalis; A, adenomyosis; NS, not significant.

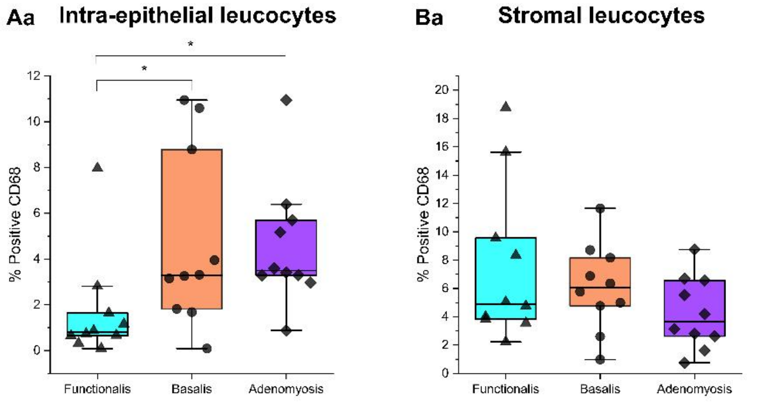

Ab

Region	% positive CD68 Median (range)	P value
Functionalis n = 10	0.81 (0.09 – 8.0)	[a] p < 0.05 [b] p < 0.05
Basalis n = 10	3.29 (0.09 – 10.95)	[a] p < 0.05
Adenomyosis n = 10	3.34 (0.88 – 6.40)	[b] p < 0.05

NS, not significant.
[a] Adenomyosis functionalis vs matched basalis.
[b] Adenomyosis lesion vs matched functionalis.

Bb

Region	% positive CD68 Median (range)	P value
Functionalis n = 10	4.91 (2.23 – 18.77)	NS
Basalis n = 10	6.07 (0.98 – 11.65)	NS
Adenomyosis n = 10	3.67 (0.75 – 8.75)	NS

C

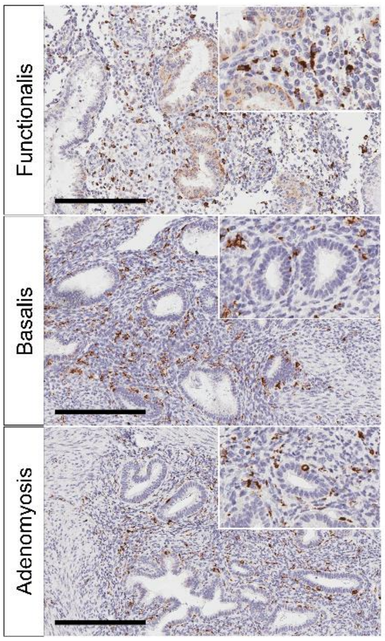

\

Figure S7. CD68+ macrophage distribution in adenomyosis lesions and matched eutopic endometrial functionalis and basalis endometrium. Aa: Boxplot and Ab: tabulated summary of percentage of CD68+ intraepithelial leucocytes in endometrial functionalis, basalis, and adenomyosis lesions. Ba: Boxplot and Bb: tabulated summary of percentage of CD68+ stromal leucocytes in endometrial functionalis, basalis, and adenomyosis lesion. The middle line represents the median value and whiskers denote minimum and maximum values. C: Representative photomicrographs of immunostaining of CD68 in endometrial functionalis, basalis, and matched adenomyosis lesions. Positive staining appears brown. Magnification 400 x. Scale bar 60 µM. NS, not significant.

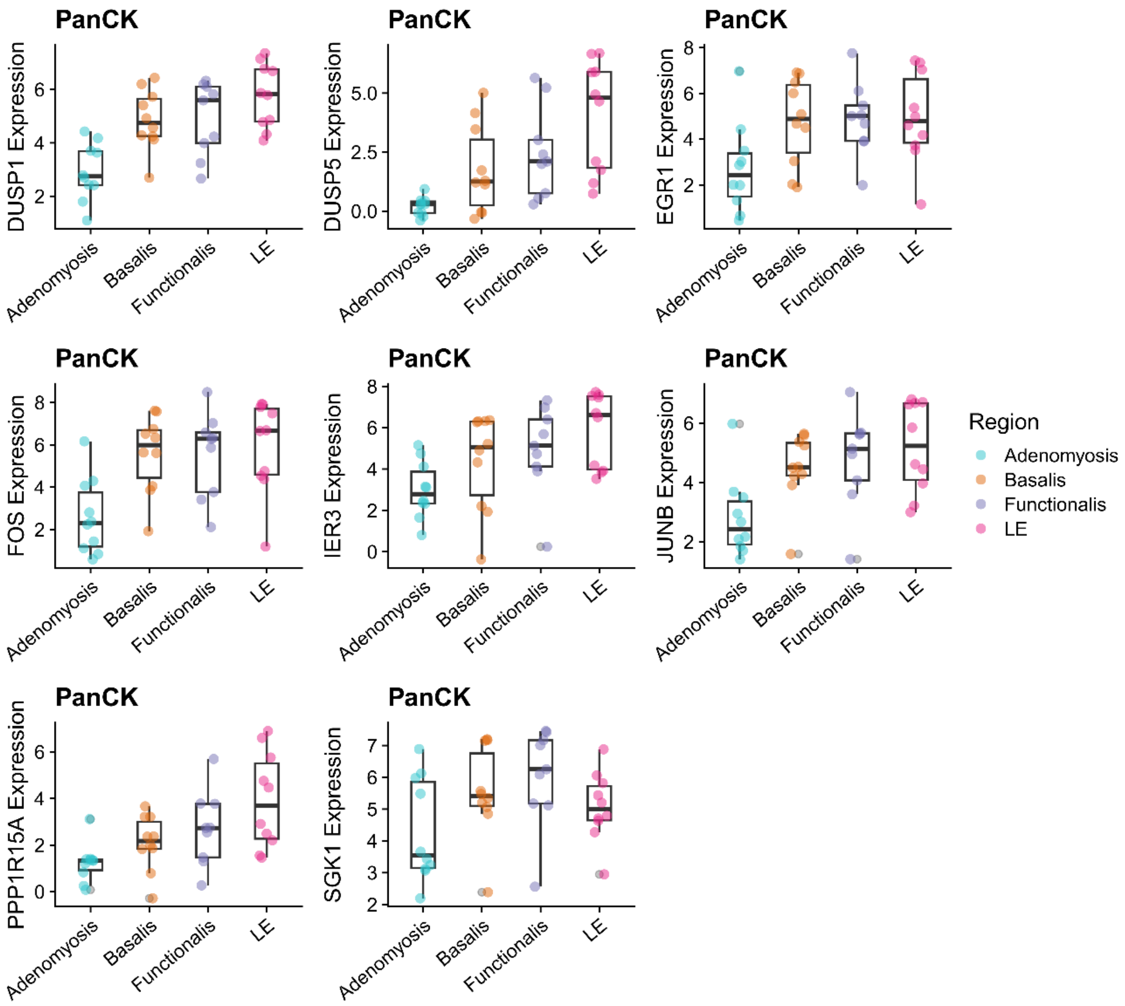

Figure S8. Boxplots of key differentially expressed gene (DEG) expression (DUSP1, DUSP5, EGR1, FOS, IER3, JUNB, PPP1R15A, SGK1) in adenomyosis lesion epithelium, eutopic endometrial basalis epithelium, eutopic endometrial functionalis epithelium, and luminal epithelium; median and range shown.

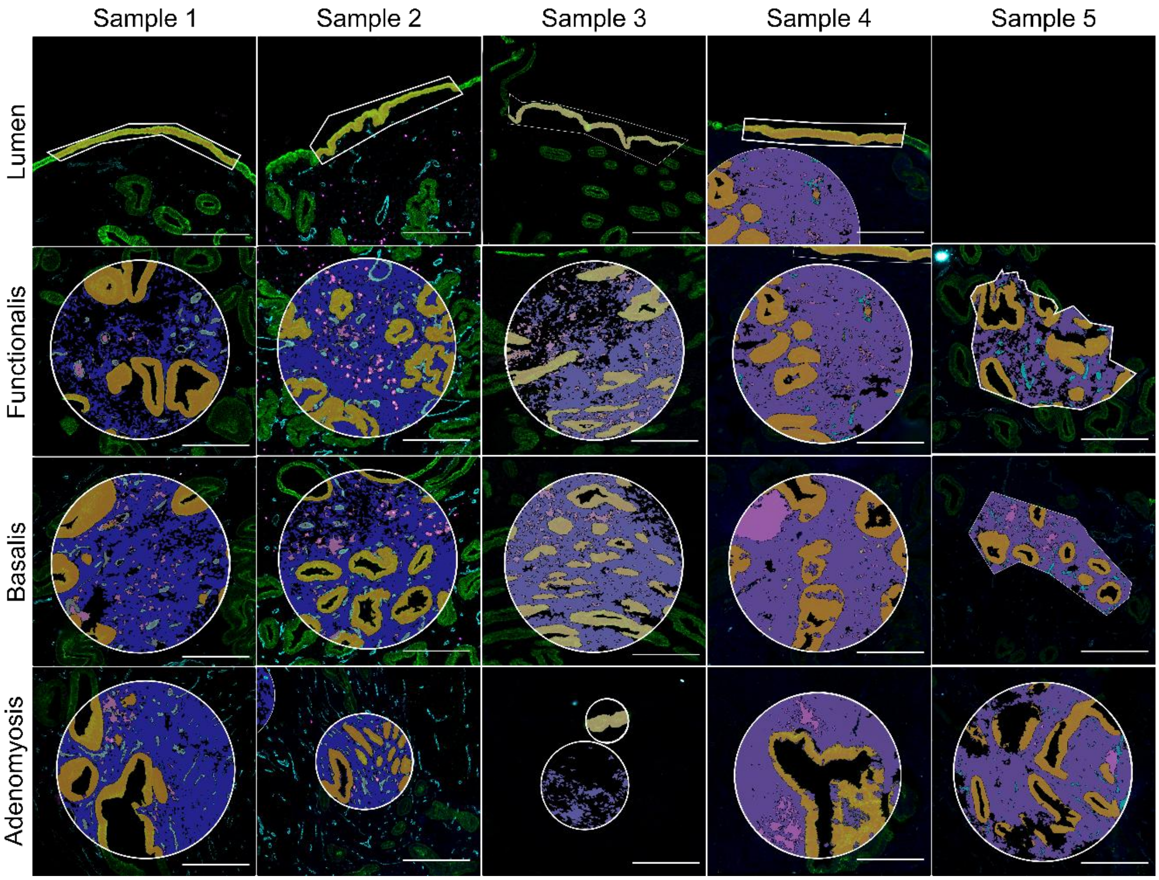

Figure S9. Digital image of region of interest (ROI) selected using the GeoMx digital spatial profiler (lumen, functionalis, basalis, and adenomyosis lesion) for patient samples 1-5. Note there was no luminal epithelium present in sample 5. Sample 4 images are also presented in Figure 8, in the latter they appear schematically to illustrate the types of regions selected for each biopsy, this reuse is intentional. Magnification x100. Scale bar in all images 200 μM.

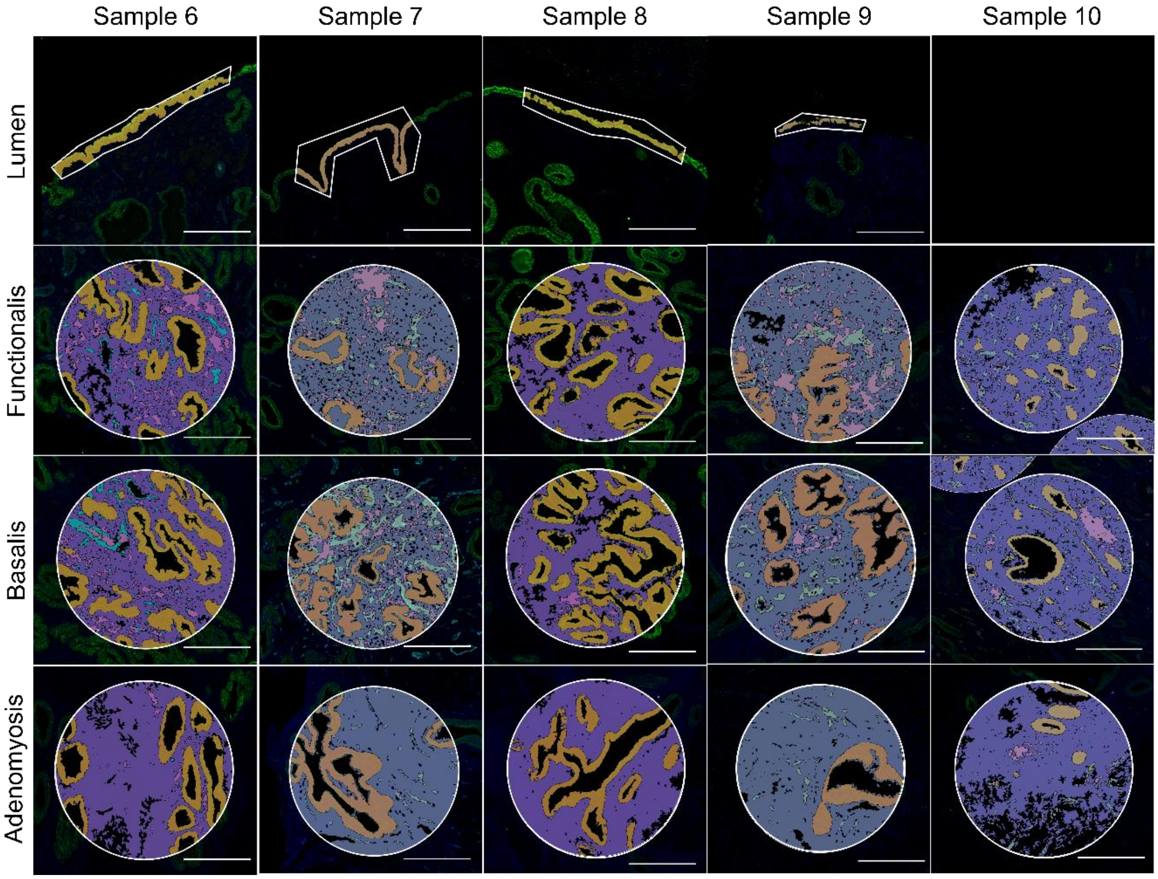

Figure S10. Digital image of region of interest (ROI) selected using the GeoMx digital spatial profiler (lumen, functionalis, basalis, and adenomyosis lesion) for patient samples 6-10. Note there was no luminal epithelium present in sample 10. Magnification x100. Scale bar in all images 200 µM.

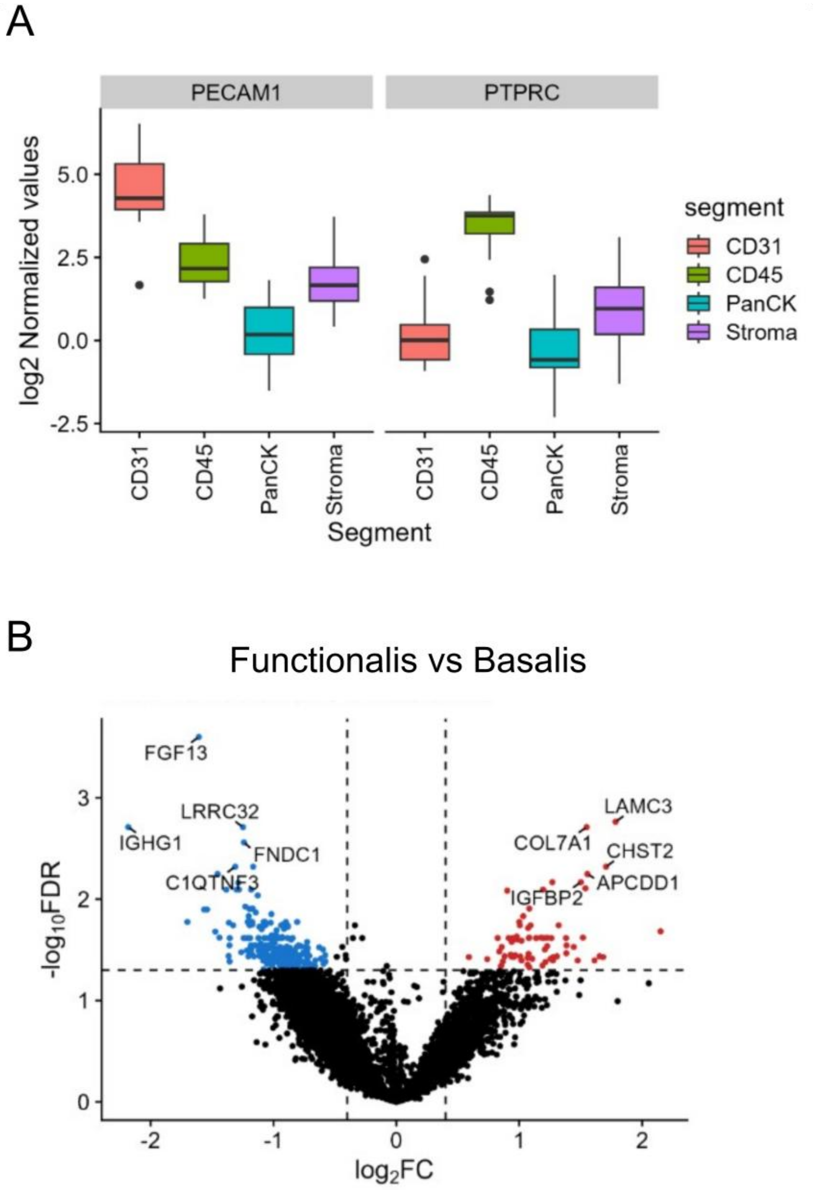

Fig. S11. Validation of cell type specificity and tissue-level expression patterns. (A) Box plots showing expression of representative marker genes across segmented populations. PECAM1, involved in platelet adhesion, was upregulated in CD31+ endothelial cell segments, and PTPRC, involved in T- and B-cell signaling, was upregulated in CD45+ immune cell segments as expected. (B) Volcano plot illustrating differentially expressed genes identified from “mock bulk” RNA-seq data generated by combining all cellular segments. Comparison of functionalis and basalis tissues revealed numerous DEGs with known biological relevance to each endometrial region.

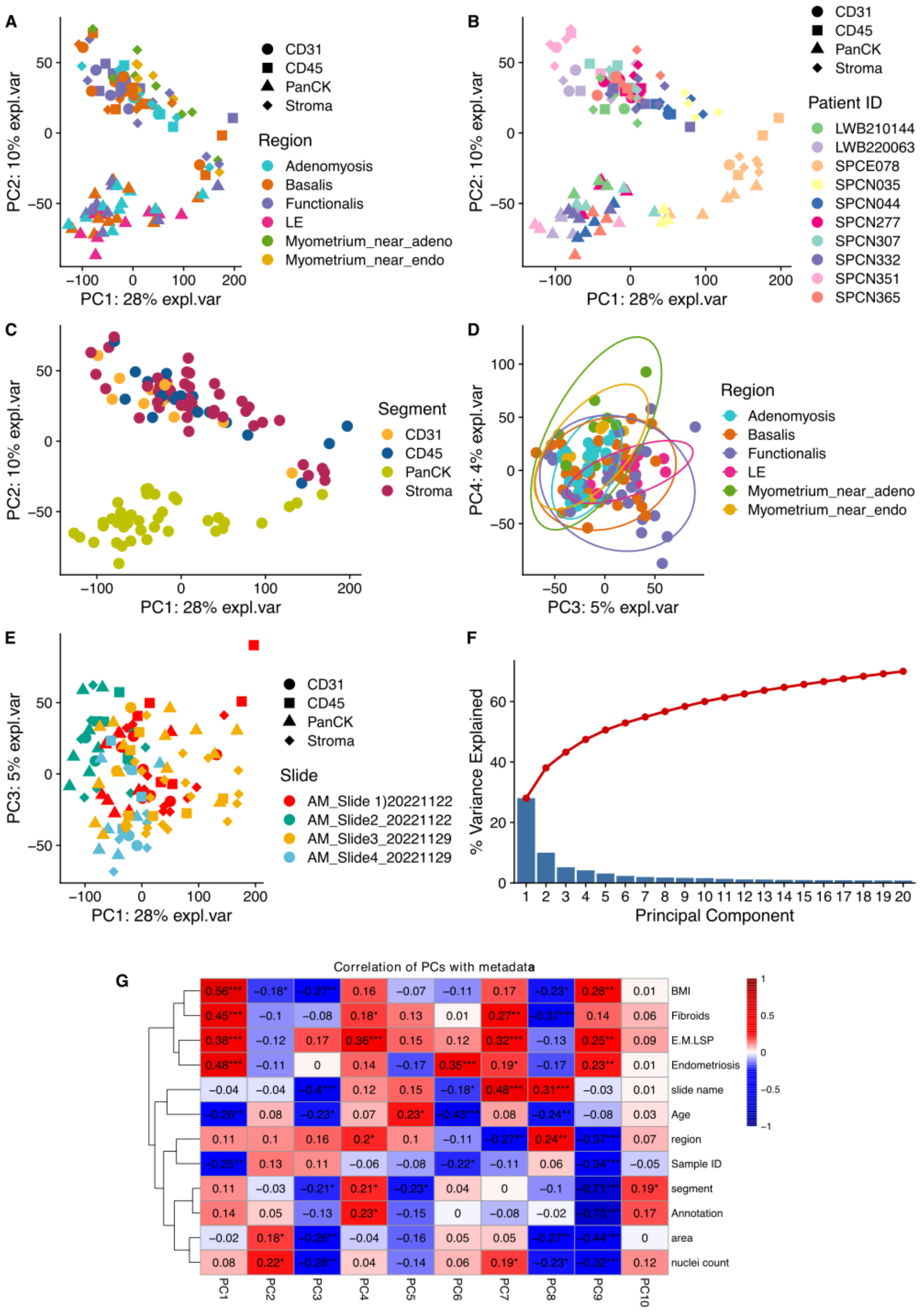

Figure S12: Data exploration and dimensionality reduction analyses. (A–E) Principal component analysis (PCA) of the normalized spatial transcriptomics data, colored by region (A), patient ID (B), segment (C), or slide (E). In panel (D), samples are displayed using PC3 and PC4 (selected due to correlation with annotation), with 95% confidence

ellipses illustrating the distribution of each anatomical region. Shapes in panels (A–E) denote the annotated segment (CD31, CD45, PanCK, or stroma). Each point represents an individual sample. (F) Scree plot demonstrating the cumulative variance explained with each additional PC. (G) Eigen correlation heatmap summarising the correlation between principal components and selected metadata variables, including patient factors: Sample ID (patient ID), age, BMI, fibroid and endometriosis status, cycle subphase status (E.M. LSP); technical factors: area, slide, nuclei count; factors of interest: annotation, segment, group. The colour of each cell encodes the strength and direction of the correlation (blue, negative; red, positive), with intensity reflecting the magnitude.

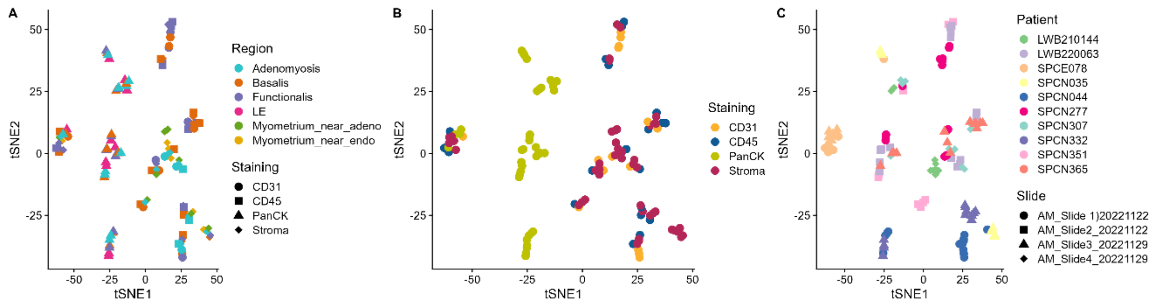

Figure S13: t-SNE visualisation of normalised spatial transcriptomics data.
(A–C) Two-dimensional t-distributed stochastic neighbour embedding (t-SNE) projection of the normalised spatial transcriptomics data, coloured by region (A), segment (B), or patient of origin (C). Shapes in panels (A) and (C) denote the staining segment or slide, respectively. The plots illustrate the global structure of the dataset and highlight grouping of ROIs primary by cell type segment, though also demonstrate technical factors such as patient grouping.

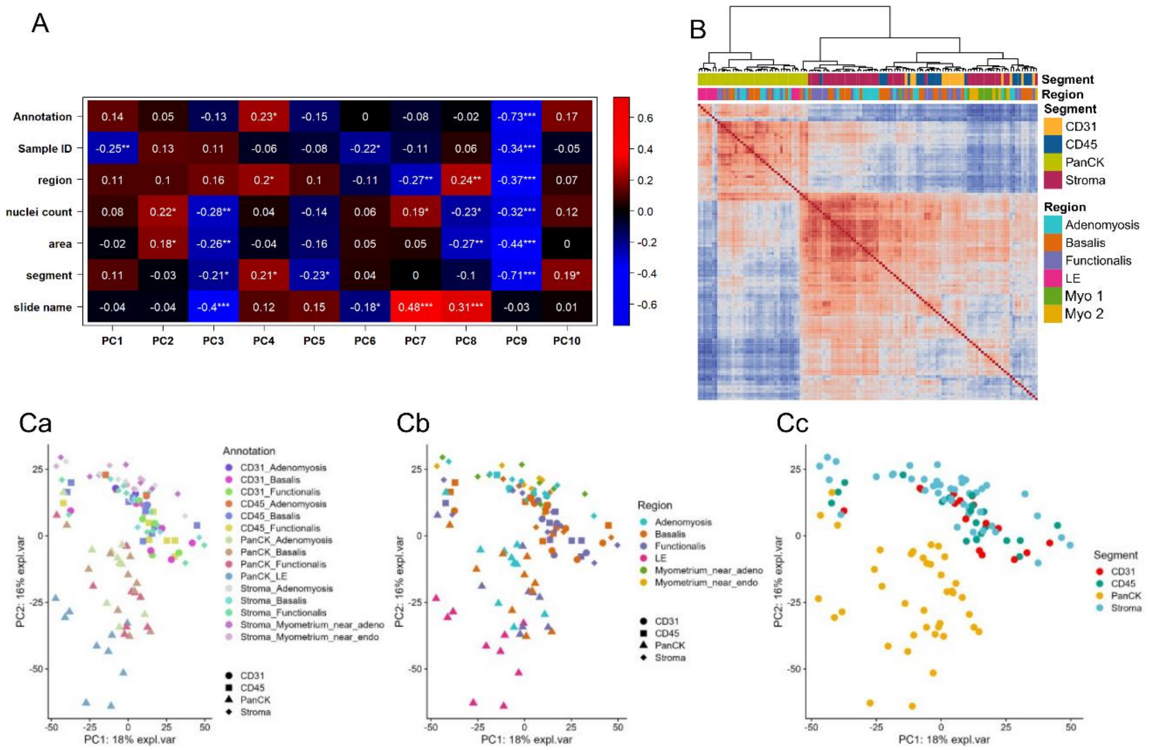

Figure S14. Data exploration and dimensionality reduction analyses. (A) Eigencor plot depicting the correlation between principal components and key variables, including sample, region, area, cell segment, and slide. The colour of each cell encodes the strength and direction of the correlation (blue, negative; red, positive), with intensity reflecting the magnitude. (B) Hierarchical clustering of Pearson correlation coefficients, visualised as a heatmap, illustrating the similarity between samples based on their expression profiles. (Ca-Cc) Principal component analysis (PCA) performed using the list of differentially expressed genes (DEGs) identified across all comparisons. The original dataset was subset by this DEG list prior to PCA. All three panels display PC1 versus PC2.

Table S1. Comparison of number of significant differentially expressed genes (DEGs) between adenomyosis lesions and matched endometrial subregions.

	**Endometrial region**		
**Segment**	**Basalis**	**Functionalis**	**Lumen**
Epithelial	2	86	1647
Stromal	14	34	NA
Immune	44	85	NA
Endothelial	59	77	NA

Table S2. The most highly down-regulated genes in adenomyosis lesion epithelium compared to endometrial basalis epithelium

**Gene**	**Description**	**NCBI gene ID**	**Adenomyosis vs basalis**	
			**Log FC**	**Adj p value**
**DOWN**				
FOS	Fos proto-oncogene, AP-1 transcription factor subunit	2353	-2.994	0.007
DUSP1	Dual specificity phosphatase 1	1843	-1.946	0.01

Table S3. The most highly up- and down-regulated genes in adenomyosis lesion epithelium compared to endometrial functionalis epithelium

Gene	Description	NCBI gene ID	Adenomyosis vs functionalis	
			**Log FC**	**Adj p value**
**DOWN**				
FOS	Fos proto-oncogene, AP-1 transcription factor subunit	2353	-2.982	0.002
NR4A2	Nuclear receptor subfamily 4 group A member 2	4929	-2.566	0.002
DUSP1	Dual specificity phosphatase 1	1843	-2.051	0.002
MT2A	Metallothionein 2A	4502	-2.256	0.002
BTG2	BTG anti-proliferation factor 2	7832	-1.882	0.002
**UP**				
SLC27A6	Solute carrier family 27 member 6	28965	1.77	0.005
IFITM1	Interferon induced transmembrane protein 1	8519	1.603	0.004
SLC25A35	Solute carrier family 25 member 35	399512	2.149	0.001
CD74	CD74	972	1.996	0.001
MMP7	Matrix metallopeptidase 7	4316	3.205	0.001

Table S4. The most highly up and down-regulated genes in adenomyosis lesion epithelium compared to luminal epithelium

Gene	Description	NCBI gene ID	Adenomyosis vs luminal epithelium	
			**Log FC**	**Adj p value**
**DOWN**				
CXCL1	C-X-C motif chemokine ligand 1	2919	-4.902	<0.0001
RRAD	Ras related glycolysis inhibitor and calcium channel regulator	6236	-4.8	<0.0001
CRISP3	Cysteine rich secretory protein 3	10321	-4.489	<0.0001
C6orf118	Chromosome 6 open reading frame 118	168090	-3.011	<0.0001
CCDC187	Coiled-coil domain containing 187	399693	-2.788	<0.0001
**UP**				
RANGRF	RAN guanine nucleotide release factor	29098	1.987	<0.0001
ALDH1A1	Aldehyde dehydrogenase 1 family member A1	216	2.314	<0.0001
SLC25A35	Solute carrier family 25 member 35	399512	2.368	<0.0001
FAM169A	Family with sequence similarity 169 member A	26049	2.311	<0.0001
SLC47A1	Solute carrier family 47 member 1	55244	1.956	<0.001

Table S5. The most highly up- and down-regulated DEGs in the stroma of adenomyosis lesions compared to the endometrial basalis stroma.

Gene	Description	NCBI gene ID	Adenomyosis lesion stroma vs basalis stroma	
			**Log FC**	**Adj P value**
**DOWN**				
FOS	Fos proto-oncogene, AP-1 transcription factor subunit	2353	-4.267	<0.0001
DUSP1	Dual specificity phosphatase 1	1843	-2.773	<0.0001
JUNB	JunB proto-oncogene, AP-1 transcription factor subunit	3726	-2.714	0.0002
ZFP36	ZFP36 ring finger protein	7538	-2.737	0.0003
EGR1	Early growth response 1	1958	-3.027	0.0011
**UP**				
ACTG2	Actin gamma 2, smooth muscle	72	2.864	0.0178

Table S6 The most highly up and down-regulated DEGs in the stroma of adenomyosis lesions compared to endometrial functionalis stroma

**Gene**	**Description**	**NCBI gene ID**	**Adenomyosis lesion stroma vs. functionalis stroma**	
			**Log FC**	**Adj P value**
**DOWN**				
FOS	Fos proto-oncogene, AP-1 transcription factor subunit	2353	-3.535	<0.0001
DUSP1	Dual specificity phosphatase 1	1843	-2.689	<0.0001
SERPINE1	Serpin family E member 1	5054	-2.891	0.0001
JUNB	JunB proto-oncogene, AP-1 transcription factor subunit	3726	-2.535	0.0001
APOE	Apolipoprotein E	348	-2.676	0.0001
**UP**				
ACTG2	Actin gamma 2, smooth muscle	72	3.723	<0.0001
MEIS2	Meis homeobox 2	4212	2.395	0.0001
MYH11	Myosin heavy chain 11	4629	2.891	0.0001
ACTA2	Actin alpha 2, smooth muscle, aorta	59	2.247	0.0012
PTGIS	Prostaglandin I2 synthase	5740	2.048	0.0012

Table S7. The most highly up and down-regulated genes in immune cells of adenomyosis lesions compared to endometrial basalis

**Gene**	**Description**	**NCBI gene ID**	**Adenomyosis lesion vs endometrial basalis**	
			**Log FC**	**Adj P value**
**DOWN**				
FOS	Fos proto-oncogene, AP-1 transcription factor subunit	2353	-3.989	0.0063
DUSP1	Dual specificity phosphatase 1	1843	-2.997	0.0017
LZTS2	Leucine zipper tumor suppressor 2	84445	-1.755	0.0369
CCN1	Cellular communication network factor 1	3491	-3.286	0.0369
SUMO2	Small ubiquitin-like modifier 2	6613	-1.118	0.0369
**UP**				
ZBTB25	Zinc finger and BTB domain containing 25	7597	2.414	0.0003
FEZF1	FEZ family zinc finger 1	389549	3.442	0.0004
HAGHL	Hydroxyacylglutathione hydrolase like	84264	2.58	0.0004
TFEC	Transcription factor EC	22797	2.598	0.0004
IGLL5	Immunoglobulin lambda-like polypeptide 5	100423062	3.401	0.0005

Table S8. The most highly up and down-regulated genes in immune cells of adenomyosis lesions compared to functionalis

Gene	Description	NCBI gene ID	Adenomyosis lesion vs endometrial functionalis	
			Log FC	Adj P value
**DOWN**				
SUMO2	Small ubiquitin-like modifier 2	6613	-1.61	0.0005
EIF5A	Eukaryotic translation initiation factor 5A	1984	-1.811	0.0005
HSPA9	Heat shock protein family A (Hsp70) member 9	3313	-1.554	0.0007
H3C2	H3 clustered histone 2	8358	-3.798	0.0045
IGFBP2	Insulin-like growth factor binding protein 2	3485	-3.116	0.0013
**UP**				
IGLL5	Immunoglobulin lambda-like polypeptide 5	100423062	4.161	<0.0001
ZBTB25	Zinc finger and BTB domain containing 25	7597	2.654	<0.0001
KLRG1	Killer cell lectin-like receptor G1	10219	2.967	0.00204
OTUD3	OTU deubiquitinase 3	23252	3.054	0.00204
CIITA	Class II major histocompatibility complex transactivator	4261	2.655	0.00454

Table S9. The most highly up and down-regulated genes in endothelial cells of adenomyosis lesions compared to endometrial basalis

Gene	Description	NCBI gene ID	Adenomyosis lesion vs endometrial basalis	
			Log FC	Adj P value
**DOWN**				
MDH2	Malate dehydrogenase 2	4191	-1.713	0.0006
COX7A2	Cytochrome c oxidase subunit 7A2	1347	-1.775	0.0101
ICMT	Isoprenylcysteine carboxyl methyltransferase	23463	-1.69	0.0176
ABCG1	ATP binding cassette subfamily G member 1	9619	-2.218	0.0181
SLC25A46	Solute carrier family 25 member 46	91137	-1.471	0.0306
**UP**				
VAX2	Ventral anterior homeobox 2	25806	2.965	0.0006
ZNF502	Zinc finger protein 502	91392	3.169	0.001
NEU3	Neuraminidase 3	10825	2.434	0.0016
MEI1	Meiotic double-stranded break formation protein 1	150365	2.036	0.0029
SPANXN3	SPANX family member N3	139067	3.115	0.0046

Table S10. The most highly up and down-regulated genes in endothelial cells of adenomyosis lesions compared to endometrial functionalis

**Gene**	**Description**	**NCBI gene ID**	**Adenomyosis lesion vs endometrial functionalis**	
			**Log FC**	**Adj P value**
**DOWN**				
GNLY	Granulysin	10578	-4.432	0.0236
MDH2	Malate dehydrogenase 2	4191	-1.601	0.0056
COX6B1	Cytochrome c oxidase subunit 6B1	1340	-1.706	0.0145
GJA1	Gap junction protein alpha 1	2697	-2.765	0.024
UQCRC1	Ubiquinol-cytochrome c reductase core protein 1	7384	-1.289	0.022
**UP**				
VAX2	Ventral anterior homeobox 2	25806	2.969	0.0023
MEI1	Meiotic double-stranded break formation protein 1	150365	2.104	0.0056
IL17RB	Interleukin 17 receptor B	55540	3.272	0.0023
ZNF766	Zinc finger protein 766	90321	2.044	0.00763
IL7	Interleukin 7	3574	2.58	0.00774

Table S11. Significantly enriched Gene Ontology (GO) terms and Reactome pathways in adenomyosis stroma compared with endometrial stroma (basalis and functionalis)

Pathway name	Adenomyosis vs basalis		Adenomyosis vs functionalis	
	GES	Adj p value	GES	Adj p value
**Geno Ontology gene sets**				
Endoplasmic reticulum protein−containing complex	-0.584	<0.0001	-0.708	<0.0001
Nucleosome	-	-	-0.706	<0.0001
Ficolin−1−rich granule	-0.63	<0.0001	-0.676	<0.0001
Protein folding	-0.607	<0.0001	-0.644	<0.0001
Ficolin−1−rich granule lumen	-0.664	<0.0001	-0.676	<0.0001
Nucleosome assembly	-	-	-0.681	<0.0001
Focal adhesion	-0.54	<0.0001	-0.559	<0.0001
Cytosolic ribosome	-0.798	<0.0001	-0.68	<0.0001
DNA packaging complex	-	-	-0.627	<0.0001
Cytoplasmic translation	-0.72	0.0062	-0.64	<0.0001
Structural constituent of ribosome	-0.681	<0.0001	-0.63	<0.0001
Ribosomal subunit	-0.667	<0.0001	-0.61	<0.0001
DNA replication−dependent chromatin assembly	-	-	-0.82	<0.0001
DNA replication−dependent chromatin organization	-	-	-0.82	<0.0001

Cytosolic large ribosomal subunit	-0.798	<0.0001	-0.683	<0.0001
Contractile fiber	0.34	0.0012	0.346	0.0033
Myofibril	0.354	0.0034	-	-
Z disc	0.418	0.004	-	-
I band	0.404	0.0052	-	-
**Reactome pathways**				
Neutrophil degranulation	-0.544	<0.0001	-0.622	<0.0001
M phase	-0.502	<0.0001	-0.626	<0.0001
Signalling by interleukins	-0.521	<0.0001	-0.586	<0.0001
Cell cycle checkpoints	-0.491	<0.0001	-0.651	<0.0001
DNA replication	-0.514	<0.0001	-0.707	<0.0001
DNA replication pre initiation	-0.549	<0.0001	-0.728	<0.0001
Rho GTPase effectors	-0.469	<0.0001	-0.617	<0.0001
Influenza infection	-0.749	<0.0001	-0.666	<0.0001
Eukaryotic translation initiation	-0.779	<0.0001	-0.698	<0.0001
Srp dependent co-translational protein targeting to membrane	-0.806	<0.0001	-0.707	<0.0001
Activated pkn1 stimulates transcription of androgen receptor regulated genes klk2 and klk3	0.793	<0.0001	-0.79	<0.0001
Cellular response to starvation	-0.733	<0.0001	-0.634	<0.0001

Response of eif2ak4 gcn2 to amino acid deficiency	-0.815	<0.0001	-0.694	<0.0001
Extracellular matrix organization	-0.436	<0.0001	-0.492	<0.0001
Degradation of the extracellular matrix	-	-	-0.515	<0.0001
Muscle contraction	0.381	<0.0001	0.344	0.0009

Table S12. Cell-type composition analysis of adenomyosis lesion stroma compared to matched endometrial functionalis stroma

	**PropMean.Stroma_Adenomyosis**	**PropMean.Stroma_Functionalis**	**PropRatio**	**Tstatistic**	**P.Value**	**FDR**
**uSMC**	0.216781949	0.029897092	7.250937525	5.67045	1E-07	1.5E-06
**Fibroblast.C7**	0.082654311	0.007024861	11.76597138	4.18957	5.4E-05	0.00038
**dS**	0.197774935	0.377041431	0.524544305	-3.7883	0.00024	0.00113
**Myeloid**	0.025394966	0.047100056	0.539170603	-2.2998	0.02323	0.0813
**Lymphoid**	0.093369184	0.183786126	0.508031732	-2.2075	0.02923	0.08185
**Endothelial.ACKR1**	0.030325627	0.008176191	3.709016619	2.01587	0.04611	0.10758
**eS**	0.130119882	0.11835913	1.099364971	1.41979	0.15833	0.31665
**PV.STEAP4**	0.138673459	0.130310034	1.064180974	1.19885	0.23301	0.40777
**Ciliated**	0.025030451	0.027090786	0.923947017	0.62001	0.53646	0.83449
**SOX9**	0.002636116	0.009607987	0.274367155	-0.4686	0.64021	0.89629
**Glandular**	0.000841383	0.002328485	0.361343546	-0.1462	0.88401	0.99729
**Other**	0.000268833	0.000350702	0.766556258	0.09962	0.92082	0.99729
**Endothelial.SEMA3G**	0.056128906	0.05892712	0.952513971	0.06342	0.94954	0.99729

**Luminal**	0	0	0	0.00<br>34	0.9972<br>9	0.99<br>729

Table S13. Cell-type composition analysis of adenomyosis lesion stroma compared to matched endometrial basalis stroma

	PropMean.Stroma_Adenomyosis	PropMean.Stroma_Basalis	PropRatio	Tstatistic	P.Value	FDR
**uSMC**	0.216781949	0.047954277	4.520596716	4.13349	6.7E-05	0.000094
**dS**	0.197774935	0.333006477	0.593907172	-2.5941	0.0107	0.07487
**Myeloid**	0.025394966	0.038592631	0.658026286	-1.5292	0.1289	0.51872
**eS**	0.130119882	0.111218049	1.169952922	1.45552	0.14821	0.51872
**Lymphoid**	0.093369184	0.135347907	0.689845793	-1.3087	0.1932	0.53334
**Endothelial.ACKR1**	0.030325627	0.014822165	2.0459647	1.21038	0.22857	0.53334
**PV.STEAP4**	0.138673459	0.156435811	0.886455968	-0.8081	0.42066	0.84132

**Glandular**	0.000841383	0.009082978	0.092632927	-0.4069	0.68486	0.99685
**SOX9**	0.002636116	0.007793166	0.338259952	-0.2654	0.79118	0.99685
**Other**	0.000268833	0	#NUM!	0.13811	0.89039	0.99685
**Endothelial.SEMA3G**	0.056128906	0.054947668	1.021497502	0.07795	0.938	0.99685
**Fibroblast.C7**	0.082654311	0.077407085	1.067787417	0.04678	0.96277	0.99685
**Ciliated**	0.025030451	0.013391785	1.869089986	-0.0356	0.97162	0.99685
**Luminal**	0	0	0	-0.004	0.99685	0.99685

Table S14. Cell-type composition analysis of adenomyosis lesion stroma compared to matched myometrium

	PropMean.Stroma_Adenomyosis	PropMean.Stroma_Myometrium_near_adeno	PropMean.Stroma_Myometrium_near_endo	PropRatio	Tstatistic	P.Value
**uSMC**	0.216781949	0.692771817	0.662393194	0.32001	-7.6667	5.7E-12
**dS**	0.197774935	0.014087477	0.02251334	11.1054	6.33383	4.6E-09
**eS**	0.130119882	0.030756178	0.012838138	6.54827	5.64516	1.2E-07
**Myeloid**	0.025394966	0.009134997	0.010112075	2.64225	3.43588	0.00082
**Fibroblast.C7**	0.082654311	0.026215283	0.01419869	4.28414	3.26127	0.00145
**Endothelial.ACKR1**	0.030325627	0.005854562	0.006045178	5.09751	2.44497	0.01598
**Lymphoid**	0.093369184	0.038219655	0.037353338	2.47113	2.31111	0.02258
**Ciliated**	0.025030451	0.012714287	0.011195278	2.098	1.83354	0.06926
**SOX9**	0.002636116	0.000172161	0.000630692	7.99997	0.65578	0.51326

**Endothelial.SEMA3G**	0.056128906	0.045964976	0.057698768	1.08991	0.44453	0.65748
**Other**	0.000268833	0	0	#NUM!	0.36426	0.71632
**Luminal**	0	0	0	#NUM!	0.06428	0.94886
**Glandular**	0.000841383	0	0	#NUM!	-0.0476	0.96212
**PV.STEAP4**	0.138673459	0.124108608	0.165021309	0.969	0.03518	0.97199

Table S15. Significantly enriched Gene Ontology (GO) terms and Reactome pathways in adenomyosis epithelium compared with endometrial epithelium (basalis, functionalis, and luminal)

**Pathway name**	**Adenomyosis vs basalis**		**Adenomyosis vs functionalis**		**Adenomyosis vs. LE**	
	**GES**	**Adj p value**	**GES**	**Adj p value**	**GES**	**Adj p value**
**Gene Ontology terms**						
Axoneme assembly	0.62x	<0.0001	0.671	<0.0001	-0.818	<0.0001
Cilium movement	0.556	<0.0001	0.583	<0.0001	-0.729	<0.0001
Cadherin binding	-0.473	<0.0001	-0.617	<0.0001	-0.628	<0.0001
Ficolin-1-rich granule lumen	-0.531	<0.0001	-0.673	<0.0001	-0.647	<0.0001
Endopeptidase complex	-0.639	<0.0001	-0.706	<0.0001	-0.642	<0.0001
Response to unfolded protein	-0.603	<0.0001	-0.627	<0.0001	-0.566	<0.0001
Focal adhesion	-0.418	<0.0001	-0.553	<0.0001	-0.486	<0.0001
Cell-substrate junction	-0.409	<0.0001	-0.547	<0.0001	-0.547	<0.0001
Cilium organization	0.412	<0.0001	0.459	<0.0001	-0.608	<0.0001
Microtubule bundle formation	0.554	<0.0001	0.59	<0.0001	-0.755	<0.0001
Response to topologically incorrect protein	-0.578	<0.0001	-0.583	<0.0001	-0.522	0.0004

Zinc ion homeostasis	-	-	-0.786	<0.0001	-0.862	<0.0001
Motile cilium	0.435	<0.0001	0.465	<0.0001	-0.698	<0.0001
Ciliary plasm	0.556	<0.0001	0.546	<0.0001	-0.741	<0.0001
Axoneme	0.558	<0.0001	0.543	<0.0001	-0.744	<0.0001
Extracellular matrix structural constituent	-	-	-0.487	<0.0001	-0.548	0.0009
Collagen-containing extracellular matrix	-	-	-	-	-0.512	<0.0001
**Reactome pathways**						
Neutrophil degranulation	-0.426	<0.0001	-0.52	<0.0001	-0.611	<0.0001
Asparagine n linked glycosylation	-0.545	<0.0001	-0.571	<0.0001	-	-
Cellular response to chemical stress	-0.502	<0.0001	-0.611	<0.0001	-	-
Signalling by interleukins	-0.381	0.0001	-0.496	<0.0001	-	-
Diseases of signal transduction by growth factor receptors and second messengers	-0.423	<0.0001	-0.497	<0.0001	-0.457	0.0018
Transport to the golgi and subsequent modification	-0.066	<0.0001	-0.594	<0.0001	-	-
ER to golgi anterograde transport	-0.527	<0.0001	-0.612	<0.0001	-	-

Metallothioneins bind metals	-0.904	0.0002	-0.96	<0.0001	-0.983	<0.0001
Platelet activation signalling and aggregation	-	-	-0.495	<0.0001	-	-
Response to metal ions	-0.846	0.0007	-0.923	<0.0001	-	-
Extracellular matrix organization	-	-	-0.438	<0.0001	-0.532	<0.0010
Collagen biosynthesis and modifying enzymes	-	-	-0.588	<0.0001	-	-

Table S16. Cell-type composition analysis of adenomyosis lesion epithelium compared to matched endometrial basalis epithelium

	PropMean.PanCK_Adenomyosis	PropMean.PanCK_Basalis	PropRatio	Tstatistic	P.Value	FDR
**Ciliated**	0.159599731	0.088548063	1.802407936	3.05125	0.00282	0.03948
**Glandular**	0.156184494	0.312760335	0.499374367	-2.1025	0.03765	0.26355
**Myeloid**	0.029860441	0.021535178	1.386589024	1.4381	0.15307	0.71432
**Fibroblast.C7**	0.017559697	0.003714981	4.726725541	1.04944	0.29614	0.84384
**uSMC**	0.044663812	0.016461899	2.713162753	1.03808	0.30137	0.84384
**Lymphoid**	0.11200512	0.088422246	1.266707485	0.84029	0.40246	0.87258
**Other**	0.010574554	0.005647738	1.872351979	0.78115	0.43629	0.87258
**SOX9**	0.239765873	0.238101367	1.006990744	0.63601	0.52601	0.92052
**dS**	0.037341437	0.043799094	0.85256186	-0.3927	0.69522	0.99047
**Luminal**	0	0.001357656	0	-0.2901	0.77226	0.99047
**Endothelial.SEMA3G**	0.062160037	0.06069291	1.024172958	0.17497	0.86141	0.99047

**eS**	0.055023503	0.05152873	1.067821839	-0.1069	0.91507	0.99047
**Endothelial.ACKR1**	0.030423009	0.028891615	1.05300479	0.06219	0.95052	0.99047
**PV.STEAP4**	0.044838291	0.038538188	1.163476892	-0.012	0.99047	0.99047

Table S17. Cell-type composition analysis of adenomyosis lesion epithelium compared to matched endometrial functionalis epithelium

	PropMean.PanCK_Adenomyosis	PropMean.PanCK_Functionalis	PropRatio	Tstatistic	P.Value	FDR
**Ciliated**	0.159599731	0.064175759	2.486916142	4.23093	4.6E-05	0.00065
**Other**	0.010574554	0	#NUM!	3.91073	0.00015	0.00108
**Glandular**	0.156184494	0.34090955	0.458140566	-2.6829	0.00836	0.03899
**Myeloid**	0.029860441	0.016247165	1.837886246	2.14548	0.03398	0.11892
**SOX9**	0.239765873	0.21306185	1.125334607	1.79333	0.0755	0.21141
**Endothelial.ACKR1**	0.030423009	0.009809655	3.101333134	1.55353	0.123	0.24844
**dS**	0.037341437	0.06103999	0.61175365	-1.5363	0.12717	0.24844
**uSMC**	0.044663812	0.006342628	7.041846692	1.47849	0.14196	0.24844
**Fibroblast.C7**	0.017559697	0	#NUM!	1.21196	0.22797	0.35462
**Luminal**	0	0.005060112	0	-0.4891	0.62569	0.86011
**Endothelial.SEMA3G**	0.062160037	0.058666028	1.059557628	0.41925	0.6758	0.86011

**PV.STEAP4**	0.044838291	0.04599273	0.974899539	-0.1122	0.91084	0.98958
**Lymphoid**	0.11200512	0.115082364	0.973260514	0.03408	0.97287	0.98958
**eS**	0.055023503	0.06361217	0.864983906	0.01308	0.98958	0.98958

Table S18. Cell-type composition analysis of adenomyosis lesion epithelium compared to matched endometrial luminal epithelium

	PropMean.PanCK_Adenomyosis	PropMean.PanCK_LE	PropRatio	Tstatistic	P.Value	FDR
**Luminal**	0	0.139494033	0	-7.5678	9.5E-12	1.3E-10
**Ciliated**	0.159599731	0.408532095	0.390666323	-6.5439	1.7E-09	1.2E-08
**SOX9**	0.239765873	0.024125886	9.938116968	6.38479	3.6E-09	1.7E-08
**Other**	0.010574554	0.000390927	27.04994298	4.04829	9.3E-05	0.00033
**Endothelial.ACKR1**	0.030423009	0.001316368	23.11132365	2.29661	0.02342	0.06558
**PV.STEAP4**	0.044838291	0.021753494	2.061199502	2.08085	0.03963	0.09247
**Lymphoid**	0.11200512	0.07764336	1.442558911	1.95108	0.05344	0.10688
**Fibroblast.C7**	0.017559697	1.00154E-18	1.75326E+16	1.35345	0.17852	0.31241
**Endothelial.SEMA3G**	0.062160037	0.047409582	1.31112814	1.15035	0.25235	0.37621
**eS**	0.055023503	0.030869834	1.782435991	1.0938	0.27629	0.37621
**uSMC**	0.044663812	0.014862135	3.005208302	1.05064	0.29559	0.37621
**Myeloid**	0.029860441	0.031752166	0.940422186	0.32338	0.74698	0.87148

**dS**	0.037341437	0.028384873	1.315540047	0.13361	0.89394	0.90958
**Glandular**	0.156184494	0.173465248	0.90037916	0.11381	0.90958	0.90958

Table S19: Significantly enriched Gene Ontology (GO) terms and Reactome pathways in adenomyosis immune cells compared with endometrial immune cells (basalis and functionalis).

Pathway name	Adenomyosis vs basalis		Adenomyosis vs functionalis	
	**GES**	**Adj p value**	**GES**	**Adj p value**
**Geno Ontology gene sets**				
Nucleosome	-0.489	<0.0001	-0.722	<0.0001
Nucleosome assembly	-0.511	<0.0001	-0.711	<0.0001
DNA replication-dependent chromatin assembly	-0.712	0.0003	-0.893	<0.0001
DNA replication-dependent chromatin organisation	-0.712	0.0003	-0.893	<0.0001
Protein-DNA complex	-		-0.62	<0.0001
Protein-DNA complex assembly	-0.358	0.0017	-0.638	<0.0001
DNA packaging complex	-0.418	0.0002	0.501	<0.0001
Nucleosome organisation	0.476	<0.0001	0.5	<0.0001
Olfactory receptor activity	0.475	<0.0001	0.491	<0.0001
Detection of chemical stimulus involved in sensory perception of smell	0.467	<0.0001	-0.722	<0.0001
Detection of chemical stimulus	-0.489	<0.0001	-0.711	<0.0001
**Reactome pathways**				
M phase	-	-	-0.591	<0.0001
DNA replication	-	-	-0.708	<0.0001
DNA replication pre initiation	-0.499	<0.0001	-0.741	<0.0001
Cell cycle checkpoints	-	-	-0.605	<0.0001

Mitotic prophase	-0.53	<0.0001	-0.747	<0.0001
Rho GTPase effectors	-	-	-0.573	<0.0001
Transcriptional regulation of granulopoiesis	-0.647	<0.0001	-0.816	<0.0001
Senescence-associated secretory phenotype SASP	-0.607	<0.0001	-0.77	<0.0001
Oestrogen-dependent gene expression	-0.549	<0.0001	-0.662	<0.0001

Table S20. Significantly enriched Gene Ontology (GO) terms and Reactome pathways in adenomyosis endothelium compared with endometrial endothelium (basalis and functionalis)

**Pathway name**	**Adenomyosis vs basalis**		**Adenomyosis vs functionalis**	
	**GES**	**Adj p value**	**GES**	**Adj p value**
**Geno Ontology gene sets**				
Nucleosome assembly	-	-	-0.601	<0.0001
Ficolin-1-rich granule lumen	-0.501	<0.0001	-0.571	<0.0001
Nucleosome	-	-	-0.563	<0.0001
Inner mitochondrial membrane protein complex	-0.535	<0.0001	-0.547	<0.0001
Endoplasmic reticulum protein-containing complex	-0.57	<0.0001	-0.548	<0.0001
Nucleosome organisation	-	-	-0.527	<0.0001
Protein-DNA complex assembly	-	-	-0.495	<0.0001
Protein-DNA complex subunit organisation	-	-	-0.445	<0.0001
Olfactory receptor activity	0.543	<0.0001	0.447	<0.0001
Detection of chemical stimulus involved in sensory perception of smell	0.54	<0.0001	0.443	<0.0001
**Reactome pathways**				
M phase	-0.343041109	<0.0001	-0.433166131	<0.0001
Neutrophil degranulation	-0.402209133	<0.0001	-0.409684218	<0.0001

HCMV early events	-	-	-0.616709721	<0.0001
Rho GTPase effectors	-0.307403451	0.0016	-0.454678556	<0.0001
DNA replication	-0.461550719	<0.0001	-0.540767933	<0.0001
PRC2 methylates histones and DNA	-	-	-0.722079901	<0.0001
Transcription regulation of granulopoiesis	-	-	-0.665352873	<0.0001
SIRT1 negatively regulates RNA expression	-	-	-0.717050577	<0.0001
Assembly of the ORC complex at the origin of replication	-	-	-0.700141436	<0.0001
Meiotic recombination	-	-	-0.639117085	<0.0001
HIV infection	-0.470691975	<0.0001	-0.425125541	<0.0001
SARS COV infections	-0.382488736	<0.0001	-0.339529652	<0.0001
Aspargine N-linked glycosylation	-0.433922316	<0.0001	-0.376813277	<0.0001
Olfactory signalling pathway	0.5365848	<0.0001	0.436435856	<0.0001

Table S21. Cell-type composition analysis of adenomyosis lesion immune cells compared to matched endometrial basalis immune cells

	PropMean.CD45_Adenomyosis	PropMean.CD45_Basalis	PropRatio	Tstatistic
**B.cells**	0.083752208	0.016039596	5.221590815	4.1759
**Dendritic.cells**	0.046020244	0.01314044	3.502184426	2.90683
**CD8..T.cells**	0.019382436	0.003622642	5.350358798	2.63095
**CD4..T.cells**	0.050968154	0.021389525	2.382855821	2.05264
**Monocytes**	0.042690043	0.068797277	0.620519367	-1.4049
**T.cells**	0.299872585	0.378251624	0.792785981	-1.2467
**Progenitors**	0.29580404	0.313956083	0.942182858	-0.6312
**Basophils**	0.010123728	0.018149493	0.557796716	-0.415
**NK.cells**	0.151386563	0.16665332	0.908392123	-0.2496

Table S22. Cell-type composition analysis of adenomyosis lesion immune cells compared to matched endometrial functionalis immune cells

	PropMean.CD45_Adenomyosis	PropMean.CD45_Functionalis	PropRatio	Tstatistic
**B.cells**	0.083752208	0.001817129	46.09039348	5.91546
**CD8..T.cells**	0.019382436	0	#NUM!	3.87568
**Progenitors**	0.29580404	0.496385193	0.595916324	-2.9514
**CD4..T.cells**	0.050968154	0.005401554	9.43583142	2.7205
**Dendritic.cells**	0.046020244	0.019025224	2.418906832	2.67481
**T.cells**	0.299872585	0.136361789	2.199095416	2.17811
**NK.cells**	0.151386563	0.269129267	0.562505018	-1.1009
**Monocytes**	0.042690043	0.064054543	0.666463931	-0.3469
**Basophils**	0.010123728	0.0078253	1.293717501	0.28051

Table S23. immune-epithelial signalling metrics, top 50 weighted ligand-receptor interactions

test_ligand	aupr_corrected	pearson
FAM3D	0.05603545	0.118512993
PLG	0.053696947	0.115457072
TAFA4	0.053166895	0.117002892
CXCL3	0.048706856	0.124726594
SERPINE1	0.048324785	0.105050352
TNFSF10	0.046315807	0.152526975
MMP9	0.044823195	0.112249853
NPFF	0.04461553	0.099550328
MST1	0.04438882	0.092462869
SEMA4A	0.044129811	0.10963301
AGRN	0.043680232	0.105699836
F10	0.043498659	0.102881505
SLC6A8	0.043321994	0.097548

PGF	0.042905193	0.097020469
SST	0.042679286	0.082390466
CCL3L3	0.042482245	0.106467343
ENG	0.042352093	0.097031718
PRSS3	0.041994602	0.114467239
FAM3C	0.041882199	0.092860903
IGFBP7	0.041710856	0.095414874
SERPINA1	0.041415395	0.091488782
NEGR1	0.040344862	0.090595566
EFNA5	0.040283785	0.104439127
EFNA2	0.040007798	0.092177325
CCN1	0.039900255	0.095177854
COL7A1	0.039803119	0.09447246
ICAM2	0.039409452	0.095888965
ANGPTL6	0.039143849	0.089341024

CCL4L2	0.039133763	0.083951353
CALM1	0.038916923	0.102006251
CFH	0.038665415	0.091716787
APOB	0.038631726	0.09966033
NAMPT	0.038539556	0.088185768
HDC	0.038408991	0.099885552
GHRL	0.038235743	0.091704538
ADAM28	0.038157723	0.097832589
CALM2	0.037959967	0.084223087
EMC1	0.037880013	0.090088903
COL24A1	0.037809228	0.090022731
GSTP1	0.037766137	0.090673752
PDGFB	0.037677824	0.101257435
WNT8A	0.037565583	0.055076294
WNT3	0.037466685	0.054411628

CNTN3	0.037463582	0.092906469
ARF1	0.037282468	0.092616268
WNT9A	0.037104114	0.053789418
LINGO4	0.036930061	0.089756354
COL6A2	0.036828533	0.090465726
FGF13	0.036776534	0.092716968
HSPG2	0.036642897	0.088367285

Table S24. Immune-epithelial signalling metrics, top 30 weighted ligand-receptor interactions

ligand	target	weight
TNFSF10	EGR1	0.152566568
TNFSF10	IER3	0.151260991
TNFSF10	SGK1	0.146641126
TNFSF10	DUSP1	0.144202861
TNFSF10	JUNB	0.14401275
TNFSF10	PPP1R15A	0.143969604
TNFSF10	DUSP5	0.143673175
MST1	VEGFA	0.143108548
TNFSF10	FOS	0.135692583
TNFSF10	VEGFA	0.112145073
MST1	EGR1	0.084609962
MST1	IER3	0.083526101
MST1	FOS	0.082391138

TNFSF10	JUND	0.082038336
MST1	SOX9	0.079021457
SERPINE1	VEGFA	0.078056849
MST1	MT1M	0.077404795
MST1	DUSP5	0.075931416
MST1	JUNB	0.074641462
MST1	PPP1R15A	0.074555028
MST1	ADM	0.074450639
MMP9	VEGFA	0.074031861
PLG	FOS	0.072338259
PLG	EGR1	0.071216837
CXCL3	VEGFA	0.069426667
MMP9	FOS	0.063259993
SEMA4A	VEGFA	0.048266884
TAFA4	FOS	0.035138832

TAFA4	VEGFA	0.0335008
FAM3D	VEGFA	0.025521259

Table S25. Endothelial-immune signalling metrics, top 50 weighted ligand-receptor interactions

test_ligand	aupr_corrected	pearson
EFNA5	0.032642922	0.071913225
CD320	0.029266728	0.03934512
JAM2	0.029266216	0.037384871
ANGPTL4	0.029090137	0.031433868
ECM1	0.028969705	0.029657423
PDGFD	0.02761285	0.028677048
PODXL	0.027299552	0.041797971
EGFL8	0.026862605	0.034474784
AGRN	0.026759686	0.067621725
S100A10	0.026376809	0.032654523
TGOLN2	0.025899272	0.032343406
NRG4	0.025707987	0.029029032
PVR	0.025287895	0.029987003

SDK2	0.025257699	0.028381328
FAT4	0.025242691	0.029787378
CD22	0.025155484	0.032538128
TIMP3	0.024824137	0.031550242
GZMB	0.024630131	0.030075953
IGF2	0.024263383	0.017129912
MUC7	0.024171217	0.031091017
LILRB2	0.02402617	0.028991571
CPAMD8	0.023676052	0.035040334
WBP1	0.02363038	0.027130547
KIRREL1	0.023540147	0.032233434
MAG	0.023537133	0.030764804
ICAM2	0.023329585	0.043644863
PLAT	0.023178112	0.055594141
SERPING1	0.023040723	0.031703536

CCN3	0.022939148	0.05519984
PAM	0.022882842	0.024431522
CIB1	0.02287892	0.030053495
CALM1	0.022757142	0.064807682
PLG	0.022515026	0.050613004
SST	0.022464604	0.062210825
SLITRK6	0.022158618	0.032179233
SEMA3G	0.021769903	0.027528379
PTPRM	0.021714047	0.036268992
GNAS	0.021533867	0.055708864
ESM1	0.021349056	0.023729251
LRRN2	0.021291449	0.022733899
GRN	0.020901465	0.046142017
ENTPD1	0.020752845	0.056818523
LAMA4	0.020741515	0.027929248

NPTX2	0.020588552	0.021315086
F10	0.020576264	0.060098942
PRL	0.020332056	0.033778392
NEO1	0.02019861	0.028467322
LAYN	0.019371486	0.031480635
COL15A1	0.01928049	0.033073127
PIGA	0.01927471	0.075868551

Table S26. Endothelial-immune signalling metrics, top 30 weighted ligand-receptor interactions

ligand	target	weight
PDGFD	MYC	0.158779702
PDGFD	FOS	0.146703367
PDGFD	DUSP1	0.139337293
ANGPTL4	FOS	0.082239296
ANGPTL4	SERPINE1	0.081390982
ANGPTL4	DKK1	0.075100495
EFNA5	FOS	0.069032948
AGRN	FOS	0.068913619
JAM2	MYC	0.025344776
PODXL	H4C8	0.025283803
JAM2	FOS	0.024388388
ECM1	MYC	0.021287885
JAM2	DUSP1	0.021132974

JAM2	SERPINE1	0.020984101
S100A10	MYC	0.019796343
EFNA5	MYC	0.019573129
PODXL	MYC	0.018725006
AGRN	MYC	0.018437829
CD320	MYC	0.01821775
JAM2	DKK1	0.016611577
JAM2	EIF5A	0.015736424
JAM2	LPXN	0.015350454
S100A10	FOS	0.015226205
ECM1	FOS	0.014258006
EGFL8	MYC	0.013862875
ECM1	SERPINE1	0.013400319
CD320	FOS	0.0126326
AGRN	SERPINE1	0.012122682

PODXL	FOS	0.011965576
EFNA5	SERPINE1	0.011879971

Table S27. Endothelial-epithelial signalling metrics, top 50 weighted ligand-receptor interactions

**test_ligand**	**aupr_corrected**	**pearson**
FAM3D	0.05603545	0.118512993
PLG	0.053696947	0.115457072
SERPINE1	0.048324785	0.105050352
TNFSF10	0.046315807	0.152526975
NPFF	0.04461553	0.099550328
MST1	0.04438882	0.092462869
SEMA4A	0.044129811	0.10963301
AGRN	0.043680232	0.105699836
F10	0.043498659	0.102881505
SLC6A8	0.043321994	0.097548
PGF	0.042905193	0.097020469
SST	0.042679286	0.082390466
SEMA3B	0.042597293	0.108874449

CCL3L3	0.042482245	0.106467343
ENG	0.042352093	0.097031718
ACE	0.042297199	0.100399016
SEMA3A	0.042168959	0.107793201
PRSS3	0.041994602	0.114467239
FAM3C	0.041882199	0.092860903
IGFBP7	0.041710856	0.095414874
SERPINA1	0.041415395	0.091488782
MYEOV	0.041339982	0.091155813
CCL18	0.04066707	0.107108464
LAMA5	0.040438543	0.095686232
CLU	0.040395616	0.094809121
NEGR1	0.040344862	0.090595566
EFNA5	0.040283785	0.104439127
CCN1	0.039900255	0.095177854

COL7A1	0.039803119	0.094472 46
MUC1	0.039714395	0.104774 311
ICAM2	0.039409452	0.095888 965
CALM1	0.038916923	0.102006 251
PTH	0.038853661	0.100925 676
APOB	0.038631726	0.099660 33
NAMPT	0.038539556	0.088185 768
SDC4	0.037996379	0.091163 772
CALM2	0.037959967	0.084223 087
EMC1	0.037880013	0.090088 903
GSTP1	0.037766137	0.090673 752
PDGFB	0.037677824	0.101257 435
FCN1	0.037574125	0.092020 279
WNT8A	0.037565583	0.055076 294
WNT3	0.037466685	0.054411 628

ARF1	0.037282468	0.092616268
LINGO4	0.036930061	0.089756354
NFASC	0.036930061	0.089756354
COL6A2	0.036828533	0.090465726
TFPI	0.036801806	0.102300493
FGF13	0.036776534	0.092716968
HSPG2	0.036642897	0.088367285

Table S28. endothelial-epithelial signalling metrics, top 30 weighted ligand-receptor interactions

**ligand**	**target**	**weight**
TNFSF10	EGR1	0.152566568
TNFSF10	IER3	0.151260991
TNFSF10	SGK1	0.146641126
TNFSF10	DUSP1	0.144202861
TNFSF10	JUNB	0.14401275
TNFSF10	PPP1R15A	0.143969604
TNFSF10	DUSP5	0.143673175
MST1	VEGFA	0.143108548
TNFSF10	FOS	0.135692583
TNFSF10	VEGFA	0.112145073
MST1	EGR1	0.084609962
MST1	IER3	0.083526101
MST1	FOS	0.082391138

TNFSF10	JUND	0.082038336
MST1	SOX9	0.079021457
SERPINE1	VEGFA	0.078056849
MST1	MT1M	0.077404795
F10	VEGFA	0.076332899
MST1	DUSP5	0.075931416
MST1	JUNB	0.074641462
MST1	PPP1R15A	0.074555028
MST1	ADM	0.074450639
PLG	FOS	0.072338259
PLG	EGR1	0.071216837
AGRN	FOS	0.068913619
SEMA4A	VEGFA	0.048266884
FAM3D	VEGFA	0.025521259
FAM3D	FOS	0.024755245

SLC6A8	SGK1	0.023523292
SERPINE1	FOS	0.023420903

Table S29. Myometrial-epithelial signalling metrics, top 50 weighted ligand-receptor interactions

test_ligand	aupr_corrected	pearson
IGFL3	0.497811402	
REN	0.062709125	0.129412132
FAM3D	0.05603545	0.118512993
PLG	0.053696947	0.115457072
TAFA4	0.053166895	0.117002892
CRH	0.052485249	0.131692561
CXCL3	0.048706856	0.124726594
SERPINE1	0.048324785	0.105050352
CLDN19	0.047442694	0.098870806
TNFSF10	0.046315807	0.152526975
MMP12	0.045345589	0.08622456
QRFP	0.044782934	0.099938466
NPFF	0.04461553	0.099550328

MST1	0.04438882	0.092462869
EPGN	0.044264713	0.107465409
SEMA4A	0.044129811	0.10963301
PSG4	0.04411134	0.094065529
AGRN	0.043680232	0.105699836
F10	0.043498659	0.102881505
SLC6A8	0.043321994	0.097548
CXCL6	0.043250626	0.102910528
VIP	0.043025758	0.100667672
PGF	0.042905193	0.097020469
SST	0.042679286	0.082390466
SEMA3B	0.042597293	0.108874449
CCL3L3	0.042482245	0.106467343
ENG	0.042352093	0.097031718
SFTPD	0.04232787	0.111412778

ACE	0.042297199	0.100399016
PTGS2	0.04224888	0.11042052
SEMA3A	0.042168959	0.107793201
PRSS3	0.041994602	0.114467239
FAM3C	0.041882199	0.092860903
IGFBP7	0.041710856	0.095414874
TMEM190	0.041673188	0.097188676
SERPINA1	0.041415395	0.091488782
MYEOV	0.041339982	0.091155813
CGA	0.040979027	0.096647638
CCL18	0.04066707	0.107108464
LAMA5	0.040438543	0.095686232
CLU	0.040395616	0.094809121
NEGR1	0.040344862	0.090595566
EFNA5	0.040283785	0.104439127

MFAP3L	0.04017273	0.091171356
EGF	0.040016639	0.138127632
EFNA2	0.040007798	0.092177325
CCN1	0.039900255	0.095177854
AMELY	0.03983881	0.098429737
COL7A1	0.039803119	0.09447246
MUC1	0.039714395	0.104774311

Table S30. Myometrial-epithelial signalling metrics, top 30 weighted ligand-receptor interactions

ligand	target	weight
TNFSF10	EGR1	0.152566568
TNFSF10	IER3	0.151260991
TNFSF10	SGK1	0.146641126
TNFSF10	DUSP1	0.144202861
TNFSF10	JUNB	0.14401275
TNFSF10	PPP1R15A	0.143969604
TNFSF10	DUSP5	0.143673175
TNFSF10	FOS	0.135692583
TNFSF10	VEGFA	0.112145073
TNFSF10	JUND	0.082038336
REN	EGR1	0.078599766
SERPINE1	VEGFA	0.078056849
REN	VEGFA	0.0768748

REN	FOS	0.074610275
CRH	VEGFA	0.07411352
PLG	FOS	0.072338259
CRH	FOS	0.07193428
PLG	EGR1	0.071216837
CXCL3	VEGFA	0.069426667
CRH	SGK1	0.067914594
CRH	JUNB	0.066338231
CRH	NR4A1	0.063589925
TAFA4	FOS	0.035138832
TAFA4	VEGFA	0.0335008
FAM3D	VEGFA	0.025521259
FAM3D	FOS	0.024755245
SERPINE1	FOS	0.023420903
PLG	VEGFA	0.020940327

SERPINE1	EGR1	0.020564735
REN	DUSP1	0.01685455

Table S31. Myometrial-immune signalling metrics, top 50 weighted ligand-receptor interactions

test_ligand	aupr_corrected	pearson
IGFL3	0.495615409	
FGF5	0.043010768	0.076577366
EFNA5	0.032642922	0.071913225
CADM4	0.031701653	0.091004381
PDCD1LG2	0.031358556	0.038283989
CD1B	0.031104306	0.035010259
EPGN	0.030910758	0.058013378
PLAU	0.030606793	0.025750578
LRRTM3	0.03032395	0.034314302
RBP3	0.030314287	0.035113342
LPA	0.029687959	0.059494748
ALOX5AP	0.029628139	0.031078494
MBL2	0.029556016	0.034464857

CD320	0.029266728	0.03934512
JAM2	0.029266216	0.037384871
BMP3	0.029200108	0.043463329
ANGPTL4	0.029090137	0.031433868
ECM1	0.028969705	0.029657423
KCNA1	0.028854099	0.032736628
GP5	0.028493386	0.03332372
SERPINA5	0.028275927	0.038376809
SIGLEC7	0.027984997	0.034434856
ADAM28	0.027667975	0.039253565
PDGFD	0.02761285	0.028677048
FGF6	0.027366794	0.030798279
LCN1	0.027316718	0.029770852
PODXL	0.027299552	0.041797971
PIP	0.02723936	0.028794515

PTPRZ1	0.027073257	0.039831<br>18
EGFL8	0.026862605	0.034474<br>784
RSPO2	0.026823139	0.031377<br>422
AGRN	0.026759686	0.067621<br>725
CLDN19	0.026589192	0.035503<br>445
CLEC2A	0.026554583	0.031932<br>193
PTH2	0.026472769	0.036524<br>939
FGF22	0.026467587	0.028590<br>679
S100A10	0.026376809	0.032654<br>523
VSTM1	0.026280366	0.033500<br>718
PRRG4	0.026133544	0.025721<br>302
SIGLEC5	0.026049727	0.030223<br>764
CEACAM5	0.026045446	0.032399<br>318
NLGN4X	0.026006952	0.031793<br>179
C6orf15	0.025957272	0.031106<br>455

LRFN5	0.025928557	0.032158982
TGOLN2	0.025899272	0.032343406
C10orf99	0.025898428	0.039700264
ADM2	0.025875437	0.037623997
NRG4	0.025707987	0.029029032
TAC4	0.025615294	0.034638976
CORIN	0.025442172	0.031982167

Table S32. Myometrial-immune signalling metrics, top 30 weighted ligand-receptor interactions

**ligand**	**target**	**weight**
PLAU	FOS	0.124803173
PLAU	MYC	0.088072063
CADM4	MYC	0.076323321
CADM4	FOS	0.069657269
EFNA5	FOS	0.069032948
FGF5	GFAP	0.064594907
PLAU	DUSP1	0.060624782
PLAU	DKK1	0.060269815
EPGN	FOS	0.044938772
RBP3	MYC	0.039232772
RBP3	SERPINE1	0.023813336
RBP3	FOS	0.021429835
EFNA5	MYC	0.019573129

PDCD1LG2	FOS	0.019533902
EPGN	MYC	0.017140162
CD1B	MYC	0.016800753
PDCD1LG2	MYC	0.01673067
FGF5	MYC	0.016561743
LRRTM3	SERPINE1	0.016505292
CD1B	FOS	0.015689112
RBP3	H3C2	0.015236257
LRRTM3	MYC	0.01519959
FGF5	FOS	0.013520063
CADM4	SERPINE1	0.012217736
EFNA5	SERPINE1	0.011879971
PDCD1LG2	SERPINE1	0.011434047
LRRTM3	FOS	0.010841678
FGF5	SERPINE1	0.010734488

EPGN	SERPINE1	0.009826588
CD1B	SERPINE1	0.009495771

Note: The following tables (Tables S33 – S41) are available as a separate excel file.

Table S33. Perturbagens and predicted modes of action identified by in silico drug screening of adenomyosis lesion epithelial cells relative to endometrial functionalis epithelial cells

Table S34. Perturbagens and predicted modes of action identified by in silico drug screening of adenomyosis lesion epithelial cells relative to endometrial basalis epithelial cells

Table S35. Perturbagens and predicted modes of action identified by in silico drug screening of adenomyosis lesion stromal cells relative to endometrial functionalis stromal cells

Table S36. Perturbagens and predicted modes of action identified by in silico drug screening of adenomyosis lesion stromal cells relative to endometrial basalis stromal cells

Table S37. Perturbagens and predicted modes of action identified by in silico drug screening of endometrial functionalis stromal cells relative to endometrial basalis stromal cells

Table S38. Perturbagens and predicted modes of action identified by in silico drug screening of adenomyosis lesion immune cells relative to endometrial functionalis immune cells

Table S39. Perturbagens and predicted modes of action identified by in silico drug screening of endometrial functionalis immune cells relative to endometrial basalis immune cells

Table S40. Perturbagens and predicted modes of action identified by in silico drug screening of adenomyosis lesion endothelial immune cells relative to endometrial functionalis endothelial cells

Table S41. Perturbagens and predicted modes of action identified by in silico drug screening of adenomyosis lesion endothelial immune cells relative to endometrial basalis endothelial cells

Table S42. Primary antibodies employed to identify specific cell types within endometrium and adenomyosis lesions and their conditions.

**Antibody / antigen**	**Species**	**Supplier**	**Clone**	**Antigen retrieval**	**Incubation**	**Final conc/dilution**
PanCK (AF488)	Mouse	Novus	NBP2-33200AF488	HIAR 20, proteinase K	Overnight 4°C	4 µg/mL
CD31 (AF647)	Mouse	Abcam	JC/70A	HIAR 20, proteinase K	Overnight 4°C	40 µg/mL
CD45 (AF594)	Mouse	Novus	2B11+PD7/26	HIAR 20, proteinase K	Overnight 4°C	10 µg/mL
CD3	Rabbit	Sino biological	CJ026-R301	HIAR 5	Overnight 4°C	1:500

CD4	Mouse	Sino biological	10400-MM23	HIAR 5	Overnight 4°C	1:250
CD8	Mouse	Sino biological	10980-MM38	HIAR 5	Overnight 4°C	1:250
CD20	Rabbit	2B Scientific	MSVA-020R	HIAR 5	Overnight 4°C	1:1000

HIAR 20 and proteinase K, heat induced antigen retrieval at 99°C for 20 minutes followed by Proteinase K (1 µg/mL ThermoFisher, AM2548) at 37 ° C for 15-min, as per NanoString RNA slide preparation protocol (84).
HIAR 5, heat induced antigen retrieval by pressure cooking for 5 min in citrate buffer pH 6.0.
